# Class A1 scavenger receptor modulates glioma progression by regulating M2-like tumor-associated macrophage polarization

**DOI:** 10.18632/oncotarget.10318

**Published:** 2016-06-29

**Authors:** Hanwen Zhang, Wenbin Zhang, Xuan Sun, Ruoyu Dang, Rongmei Zhou, Hui Bai, Jingjing Ben, Xudong Zhu, Yan Zhang, Qing Yang, Yong Xu, Qi Chen

**Affiliations:** ^1^ Department of Pathophysiology, Nanjing Medical University, Nanjing, P.R. China; ^2^ Department of Neurosurgery, Brain Hospital affiliated to Nanjing Medical University, Nanjing, P.R. China

**Keywords:** glioma, tumor-associated macrophage, class A1 scavenger receptor, macrophage polarization, heat shock protein 70

## Abstract

Macrophages enhance glioma development and progression by shaping the tumor microenvironment. Class A1 scavenger receptor (SR-A1), a pattern recognition receptor primarily expressed in macrophages, is up-regulated in many human solid tumors. We found that SR-A1 expression in 136 human gliomas was positively correlated with tumor grade (P<0.01), but not prognosis or tumor recurrence. SR-A1-expressing macrophages originated primarily from circulating monocytes attracted to tumor tissue, and were almost twice as numerous as resident microglia in glioma tissues (P<0.001). The effects of SR-A1 on glioma proliferation and invasion were assessed *in vivo* using an SR-A1-deficient murine orthotopic glioma model. SR-A1 deletion promoted M2-like tumor-associated macrophage (TAM) polarization in mice by activating STAT3 and STAT6, which resulted in robust orthotopic glioma proliferation and angiogenesis. Finally, we found that HSP70 might be an endogenous ligand that activates SR-A1-dependent anti-tumorigenic pathways in gliomas, although its expression does not appear informative for diagnostic purposes. Our findings demonstrate a relationship between TAMs, SR-A1 expression and glioma growth and provide new insights into the pathogenic role of TAMs in glioma.

## INTRODUCTION

Despite significant advances in neurosurgery and chemo-radiotherapy, malignant gliomas, the most frequent primary tumors of the central nervous system, remain resistant to conventional therapeutic strategies. Glioblastoma (GBM) patients (WHO grades III and IV) have an average survival time of approximately 1.5 years after diagnosis [1, 2]. Glioma lethality is attributable to its unchecked proliferation and invasive nature cultivated largely by the glioma microenvironment. This microenvironment is a complex structure of malignant cancer cells embedded in the vasculature and surrounded by a dynamic tumor stroma, which consists of various nonmalignant cells including fibroblasts and myeloid cells [3, 4]. Macrophages, an important cell component of the tumor stroma, are recruited and mobilized by tumor-derived factors such as IL-4, IFN-γ and IL-13 and then induced to differentiate into pro-tumorigenic tumor-associated macrophages (TAMs). TAMs can promote tumor angiogenesis, proliferation and invasion [1, 2, 5], and TAM infiltration is often associated with poor prognosis. Inhibition of TAM infiltration leads to glioma shrinkage in animal models [6, 7].

At the initial phase of tumor progression, or in non-progressing or regressing tumors, TAMs assume a classically activated phenotype characterized by a pro-inflammatory response and active antigen presentation [8]. They create an inflammatory environment that is pro-mutagenic and pro-growth. As tumors progress to a more advanced and thus more invasive stage, TAMs resemble alternatively activated macrophages (M2-like), which stimulate angiogenesis, tumor cell intra/extravasations and proliferation [9]. At metastatic sites, TAMs promote tumor cell settlement and subsequent outgrowth of metastatic lesions. Recent profiling studies showed that TAMs that arise from CCR2^+^ bone marrow-derived cells could be distinguished from resident macrophages by VCAM-1 expression. The gene expression profiles of TAMs and conventional alternatively activated macrophages largely do not overlap [10-13]. Despite rigorous attempts, the precise ontogeny and function of TAMs in solid tumors, especially in gliomas, are not well understood.

Class A1 scavenger receptor (SR-A1; also known as MSR1 and CD204) is a pattern recognition receptor involved in the progression of multiple tumors [6, 7, 14]. It is a marker for alternatively activated macrophages/microglia in glioma progression [15]. SR-A1 suppresses lung cancer progression by inhibiting MMP-9 production [16, 17] and modulates macrophage polarization in cardiovascular inflammatory microenvironments [18-20]. The present study demonstrates that SR-A1 inhibits glioma growth, invasion and angiogenesis by preventing M2-like TAM infiltration and differentiation. HSP70 is identified as a potential endogenous ligand for activating the SR-A1-linked STAT3 and STAT6 signaling cascades in the glioma microenvironment. Our results provide mechanistic insights and novel rationale for targeting the HSP70/SR-A1 axis in glioma intervention.

## RESULTS

### SR-A1 expression in macrophages/microglia is associated with glioma malignancy and prognosis

**Figure 1:**
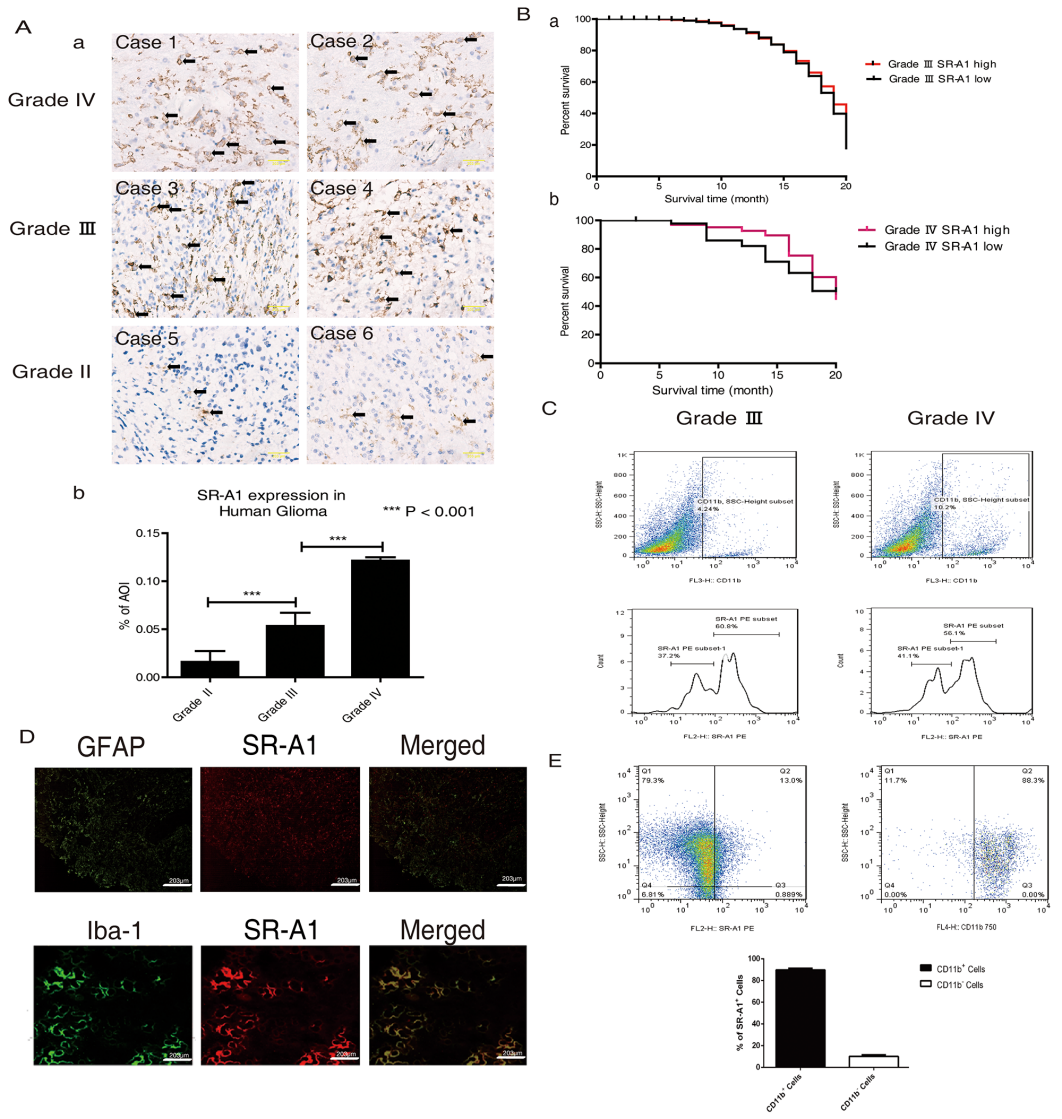
SR-A1-expressing cells influence glioma malignancy and prognosis Representative IHC staining of SR-A1 in human gliomas **Aa.** Scale bar = 100 μm. Quantitative analysis of SR-A1 expression in different human gliomas (pointed by black arrows, n = 136; ***P<0.001) **Ab.** Kaplan-Meier survival analysis of the relationship between SR-A1 expression and survival time of patients with the same glioma grade (a, grade III, n = 46; b, grade IV, n = 42) **B.** Flow cytometry analysis of SR-A1 expression in grades III and IV human gliomas **C.** CD11b^+^ dot plots were first gated for macrophages/microglia isolated from human glioma and then SR-A1 expression was examined. SR-A1^high^ macrophages/microglia was defined as higher than 10^2^. **D.** Representative immunofluorescence staining of SR-A1, glioma cell (GFAP) and macrophage (Iba-1) in human gliomas (n = 10) **E.** Flow cytometry analysis of SR-A1^+^ cells in grade IV human gliomas. SR-A1^+^ dot plots were first gated for SR-A1^+^ cells isolated from human gliomas and then CD11b^+^ microglia/macrophages were determined (n = 3; ***P<0.01).

Immunohistochemical (IHC) analysis in 136 human glioma samples showed that SR-A1 expression increased with glioma grade (Figure 1Aa & 1Ab), suggesting that SR-A1 might be associated with malignant glioma initiation and progression. Kaplan-Meier analysis revealed that GBM patients with higher SR-A1 levels had a slightly better survival rate than those with lower levels (Figure 1B). Glioma recurrence in the SR-A1^low^ expression group was more frequent than in SR-A1^high^ expression group for patients with the same GBM grade (Table 1). FACS analysis showed that 4.24% of cells in grade III gliomas and 10.2% of cells in grade IV gliomas were CD11b*^+^* macrophages. However, SR-A1^high^/CD11b^+^ macrophages accounted for 60.8% of SR-A1^+^/CD11b^+^ macrophages in grade III gliomas but only 56.1% in grade IV gliomas, reflecting decreased SR-A1^high^/CD11b^+^ macrophages in advanced malignant glioma (Figure 1C). These results suggest that SR-A1 expression is inversely associated with glioma malignance.

**Table 1:** Relationship between SR-A1 and glioma recurrence in patients with the same WHO grade

	SR-A1 high expression	SR-A1 low expression	P value
Age	51.2±4.1	46.4±3.5	0.0762
WHO Grade III	SR-A1^hi^	SR-A1^lo^	<0.001
Recurrence	1	6	
Negative	19	14	
WHO Grade IV	SR-A1^hi^	SR-A1^lo^	<0.01
Recurrence	4	6	
Negative	7	7	

SR-A1 is primarily expressed in macrophages/microglia in the brain [21]. IHC staining revealed SR-A1 co-localization with macrophages/microglia (Iba-1^+^), but not glioma cells (GFAP^+^) in human gliomas (Figure 1D). FACS measurements revealed that CD11b^+^ cells constituted about 90% of SR-A1^+^ cells in human gliomas (Figure 1E). Western blot analysis confirmed that SR-A1 was expressed in cultured macrophages but not in cultured glioma cell lines (Supplementary Figure S1B).

### SR-A1 deficiency promotes tumor growth, angiogenesis and TAM infiltration in murine orthotopic glioma

**Figure 2:**
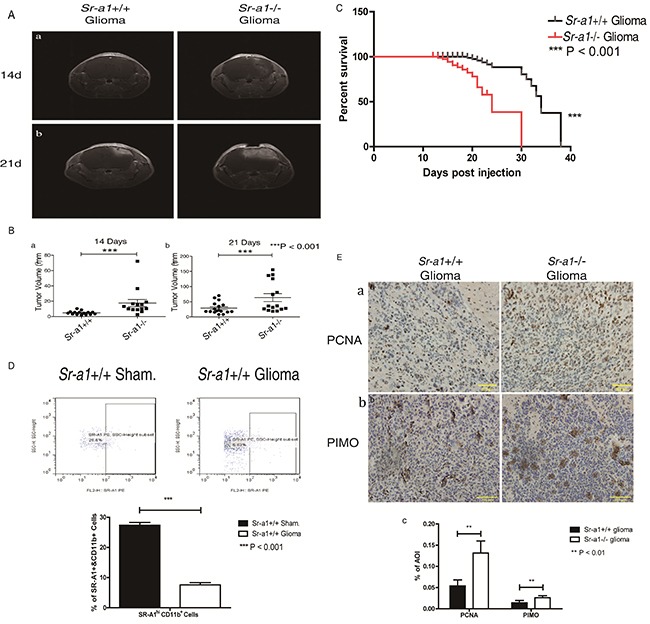
SR-A1 deficiency promotes orthotopic glioma growth and malignancy in mice Contrast-enhanced T1-weighted MRI on day 14 after intracranial implantation of GL261 murine glioma cells in mice **Aa.** Contrast-enhanced T1-weighted MRI on day 21 post implantation **Ab.** Quantitative analysis of tumor volume obtained from MRI on day 14 (pointed by write arrows, *Sr-a1*^+/+^group, n = 17; *Sr-a1*^−/−^ group, n = 14; ***P<0.001) **Ba.** Quantitative analysis of tumor volume obtained from MRI on day 21 (*Sr-a1*^+/+^group, n = 17; *Sr-a1*^−/−^ group, n = 14; ***P<0.001) **Bb.** Kaplan-Meier survival analysis of survival time for *Sr-a1*^+/+^ and *Sr-a1*^−/−^ mice with orthotopic gliomas (*Sr-a1*^+/+^group, n = 20; *Sr-a1*^−/−^ group, n = 20; ***P<0.001) **C.** Flow cytometry analysis of SR-A1 expression in normal brain and orthotopic glioma **D.** CD11b^+^ dot plots were first gated for macrophages/microglia isolated from brain and then SR-A1 expression level was examined. SR-A1 expression higher than 10^2^ was considered SR-A1^high^ macrophages/microglia. Representative IHC staining of glioma cell proliferation marker (PCNA) and glioma malignancy marker (PIMO) in murine orthotopic gliomas on day 21 post inoculation **Ea, b.** Quantitative analysis of PCNA and PIMO staining in murine orthotopic gliomas (n = 10; **P<0.01) **Ec.**

MRI analyses indicated that orthotopic gliomas in *Sr-a1*^−/−^ mice were small but visible 14 days after inoculation, whereas tumors in *Sr-a1*^+/+^ mice were barely detectable (*Sr-a1*^+/+^ glioma volume: 4.83 ± 2.35 mm^3^; *Sr-a1*^−/−^ glioma volume: 12.27 ± 3.93 mm^3^, P<0.001) (Figure 2Aa & 2Ba). On day 21 post inoculation, tumors in *Sr-a1*^−/−^ mice were significantly larger than those in *Sr-a1*^+/+^ mice (*Sr-a1*^+/+^ glioma volume: 29.0 ± 19.0 mm^3^; *Sr-a1*^−/−^ glioma volume: 63.7 ± 50.9 mm^3^, P<0.001) (Figure 2Ab & 2Bb). Histological staining of whole brain sections 21 days post inoculation showed more vigorous tumor growth in *Sr-a1*^−/−^ mice (Supplementary Figure S2). Kaplan-Meier analysis indicated that *Sr-a1*^+/+^ gliomas had better survival than *Sr-a1*^−/−^ gliomas (Figure 2C). The SR-A1^high^/CD11b^+^ macrophages were significantly decreased in murine glioma compared with normal brain (Figure 2D). *Sr-a1*^−/−^ glioma had a more robust proliferation index than *Sr-a1*^+/+^ glioma as evidenced by IHC staining of PCNA (Figure 2Ea). Hypoxia is a hallmark of glioma malignancy. PIMO staining indicated that hypoxia was greater in *Sr-a1*^−/−^ gliomas than *Sr-a1*^+/+^ gliomas (Figure 2Eb).

**Figure 3:**
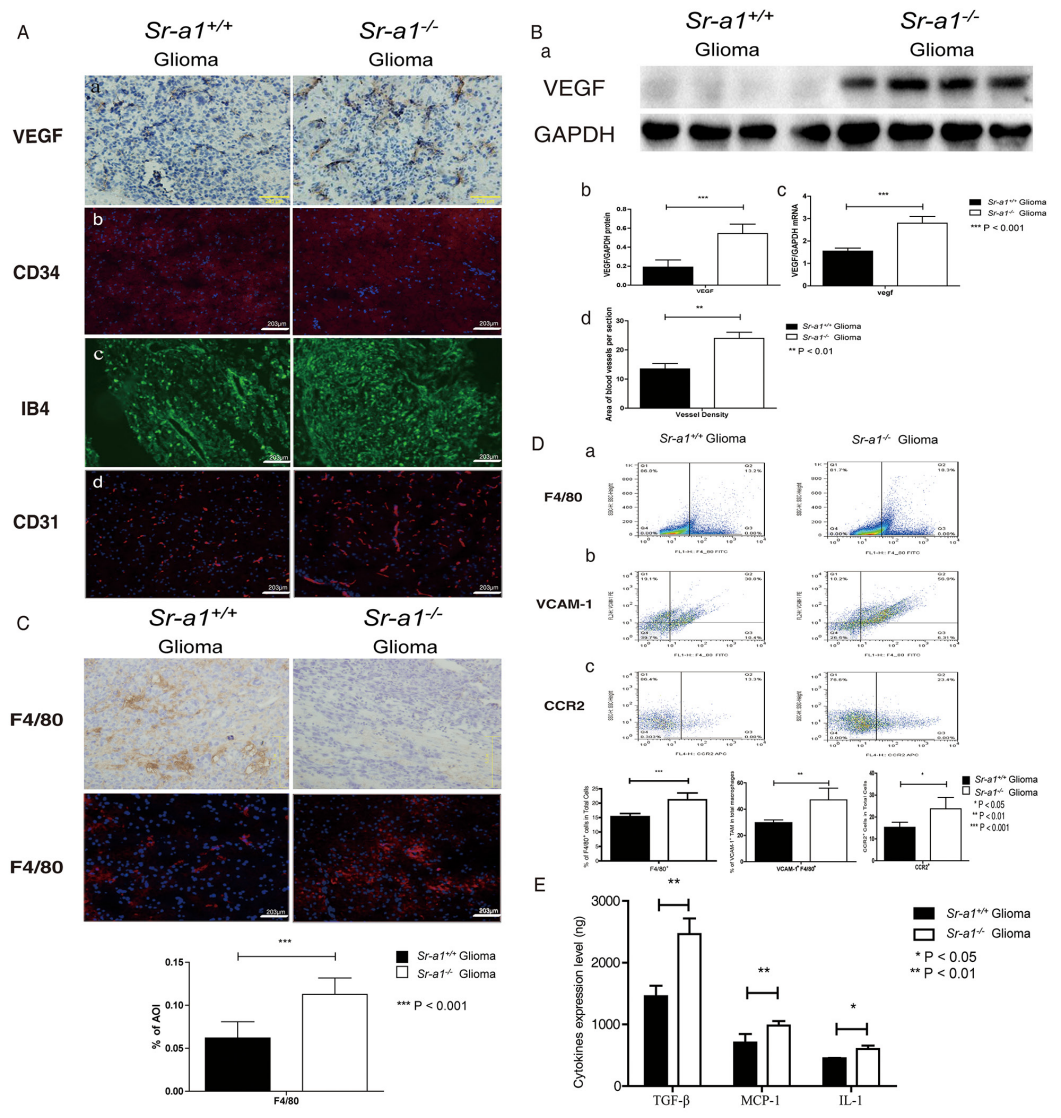
SR-A1 deficiency stimulates angiogenesis and TAM infiltration in orthotopic gliomas in mice Representative IHC and IF staining of multiple angiogenesis factors in murine orthotopic gliomas on day 21 post inoculation: VEGF **Aa**.; CD34 (CD34: Red, Nuclear: Blue) **Ab.**; IB4 (Green) **Ac.**; CD31 (CD31: Red, Nuclear: Blue) **Ad.** Representative western blot of VEGF in murine orthotopic gliomas on day 21 post inoculation (*Sr-a1*^+/+^group and *Sr-a1*^−/−^group was separated by irrelevant protein sample) **Ba.** Quantitative analysis of VEGF expression (n = 10; ***P<0.001) **Bb.** VEGF mRNA levels (n = 10; **P<0.01 **Bc.** Morphometric analysis of CD34^+^ IHC staining in murine glioma tissues at ×20 magnification **Bd.** An average of 5 to 10 random fields per mouse were analyzed using Cell P imaging software. Vessel density is defined as percentage of vessel area with tumor area. Columns, mean; bars, SD (n = 10; **P<0.01). Representative F4/80 IHC/IF staining and quantitative analysis in murine orthotopic gliomas 21 days post inoculation (F4/80: Red, Nuclear: Blue. n = 10; ***P<0.001) **C.** Flow cytometry analysis of TAM and its precursor cells in murine orthotopic gliomas **D.** F4/80^+^ dot plots represent macrophage infiltration (right quadrant) **Da.** F4/80^+^/VCAM-1^+^ dot plots represent TAM (right upper quadrant) **Db.** CCR2^+^ dot plots represent TAM precursor monocytes (right quadrant) **Dc.** (n = 10; *, P < 0.05; **P<0.01.) ELISA analysis of MCP-1, IL-1 and TGF-β levels in murine orthotopic glioma homogenates on day 21 post inoculation **E.**

Angiogenesis is required for glioma growth. Relative tumor vascular areas (CD31^+^, CD34^+^ and IB4^+^ blood vessels) were significantly increased in *Sr-a1*^−/−^ gliomas compared with *Sr-a1*^+/+^ gliomas (Figure 3Ab, 3Ac, 3Ad & 3Bd). VEGF expression was also up-regulated in *Sr-a1*^−/−^ gliomas as measured by IHC, qPCR and western blot (Figure 3Aa, 3Ba, 3Bb & 3Bc). Macrophage infiltration is critical for glioma progression. There was an increased presence of F4/80^+^ macrophages/microglia in *Sr-a1*^−/−^ gliomas compared with *Sr-a1*^+/+^ gliomas (Figure 3C & 3Da). TAMs can be identified by increased integrin receptor VCAM1 expression during tumor progression [10]. FACS measurements showed increased VCAM1-positive TAMs and CCR2^+^ TAM precursor cells in *Sr-a1*^−/−^ glioma compared with *Sr-a1*^+/+^ glioma (Figure 3Db & 3Dc), confirming that SR-A1 deletion could increase TAM infiltration. Additional pro-proliferation factors such as IL-10, TGF-β and MCP-1, which are also important TAM recruiting factors, were increased in *Sr-a1*^−/−^ gliomas compared with *Sr-a1*^+/+^ gliomas (Figure 3E). These results were corroborated by a protein chip analysis showing that multiple angiogenic and macrophage-recruiting factors such as MMP-9, VEGF and CSF were up-regulated in *Sr-a1*^−/−^ murine gliomas (Supplementary Figure S3). These data indicate that SR-A1 deletion may promote glioma progression through angiogenesis and macrophage infiltration.

### SR-A1 deficiency promotes M2-like TAM polarization in murine orthotopic glioma

**Figure 4:**
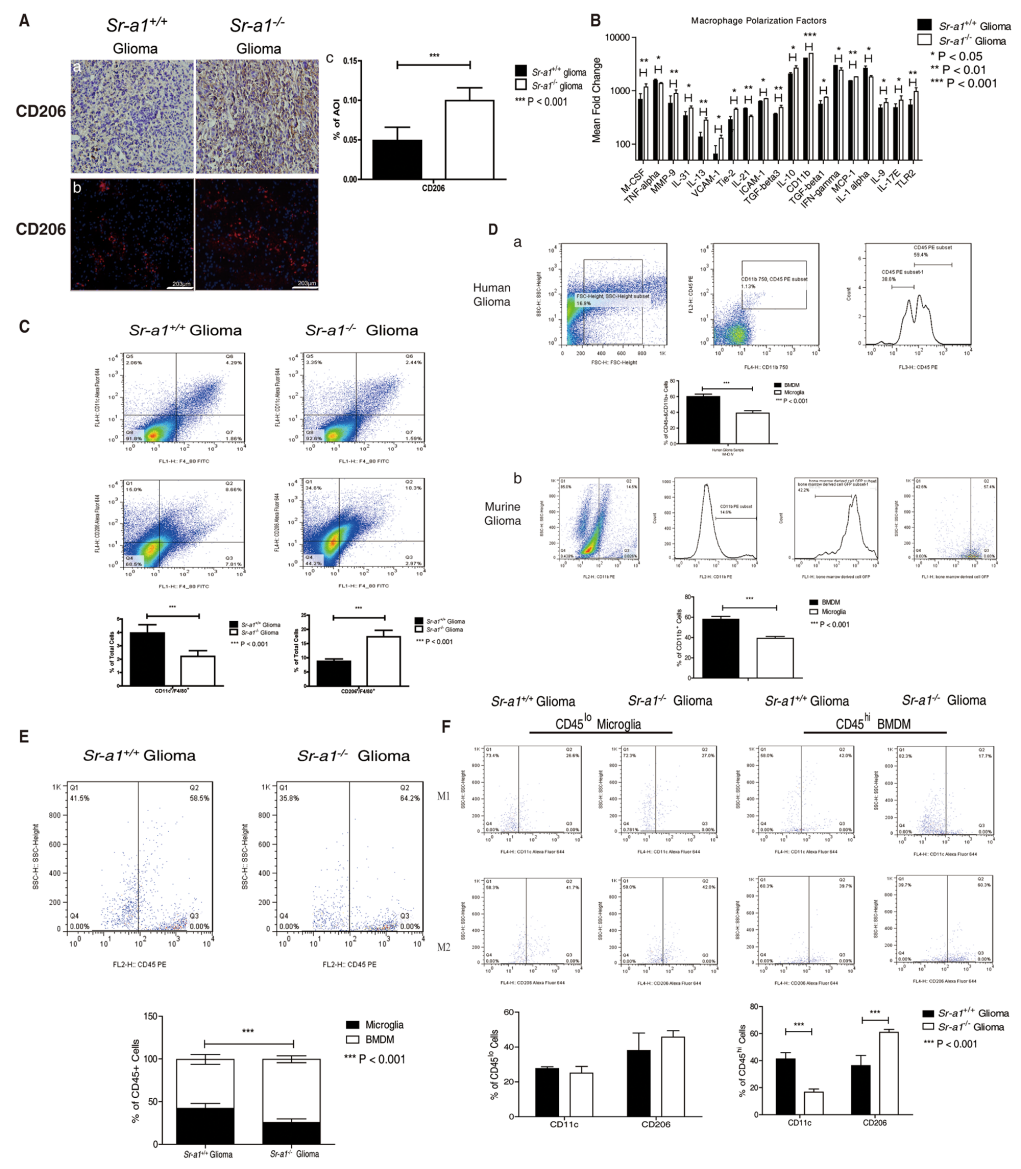
SR-A1 deficiency stimulates infiltration and polarization of M2-like BMDMs in murine orthotopic glioma Representative IHC and IF staining of CD206 in murine orthotopic gliomas on day 21 post glioma inoculation (CD206: Red, Nuclear: Blue) **Aa. & Ab.** Quantitative analysis of IHC and IF staining (n = 10; ***P<0.001) **Ac.** Protein chip assay for macrophage/microglia-related genes in murine orthotopic gliomas (n = 3; *, P<0.05) **B.** Flow cytometry analysis of macrophage/microglia polarization in orthotopic gliomas **C.** F4/80^+^/CD11c^+^ dot plots (right upper quadrant) represent M1-like microglia/macrophages. F4/80^+^/CD206^+^ dot plots (right upper quadrant) represent M2-like microglia/macrophages (n = 10; *P<0.05; **P<0.01). Representative flow cytometry analysis of BMDMs and microglia in grade IV human glioma **Da.** CD11b^+^/CD45^+^ cells are first gated for microglia/macrophages. The CD11b^+^CD45^high^ subpopulation was then gated for BMDMs. The CD11b^+^CD45^low^ subpopulation was gated for microglia. CD45 expression higher than 10^2^ was considered as CD45^high^ macrophage/microglia (n = 3; ***P<0.001). Representative flow cytometry analysis of BMDMs and microglia in *Sr-a1*^+/+^ murine orthotopic gliomas after bone marrow transplantation **Db.** Samples were first gated for CD11b^+^ macrophages/microglia; GFP-positive cells were considered BMDMs and GFP-negative cells were considered microglia (n = 6; ***P<0.001). Flow cytometry analysis of BMDMs and microglia in murine orthotopic gliomas **E.** F4/80^+^/CD45^+^ cells were first gated for microglia/macrophage. Then the CD45^high^ subpopulation (>10^2^) was gated for BMDMs (right quadrant). The CD45^lo^ subpopulation (<10^2^) was gated for microglia (left quadrant) (n = 6; **P<0.01). Flow cytometry analysis of BMDMs and microglia polarization in murine orthotopic gliomas **F.** The F4/80^+^/CD45^high^ (BMDM) and F4/80^+^CD45^low^ (microglia) subpopulations were first gated and then examined for co-expression of CD11c (M1) or CD206 (M2) (n = 6; *P<0.05).

To understand the role of SR-A1 in macrophage polarization, we examined macrophage/microglia subtypes in murine gliomas. IHC staining showed that M2-like (CD206^+^) cells were increased in *Sr-a1*^−/−^ gliomas (Figure 4A). Analyses of macrophage phenotype-specific markers using protein chips and qPCR confirmed a trend towards M2-like differentiation in macrophages/microglia in *Sr-a1*^−/−^ gliomas (Figure 4B & S4). Infiltrating cells in gliomas were further identified by FACS analyses of cell surface markers (M1-like: F4/80^+^/CD206^−^/CD11c^+^; M2-like: F4/80^+^/CD206^+^/CD11c^−^) [22]. M2-like macrophages/microglia were significantly increased and M1-like macrophages/microglia were significantly decreased in *Sr-a1*^−/−^ gliomas (M1: *Sr-a1*^+/+^: 3.96% ± 0.62%, *Sr-a1*^−/−^: 2.21% ± 0.43%; M2: *Sr-a1*^+/+^: 8.76% ± 0.84%, *Sr-a1*^−/−^: 17.4% ± 2.27%) (Figure 4C).

Glioma-associated macrophages include resident macrophages, or brain microglia, and macrophages recruited from bone marrow [23]. To differentiate the roles of these two kinds of macrophages in glioma progression, bone marrow-derived macrophages (BMDMs) were distinguished from microglia according to CD45 expression level. FACS analysis showed that the number of BMDMs (F4/80^+^/CD45^high^: 57.65% ± 3.20%) in three GBM samples was significantly higher than that of microglia (F4/80^+^/CD45^low^: 38.58% ± 3.11%) (Figure 4Da). We transplanted GFP transgenic mouse bone marrow to *Sr-a1*^+/+^ recipient mice and performed orthotopic glioma inoculations after 4 weeks of recovery. The transplanted BMDMs and resident microglia in glioma tissues were identified as CD11b^+^/GFP^+^ and CD11b^+^/GFP^−^ cells, respectively. Consistent with a previous report that CD11b-positive macrophages/microglia constitute approximately 20% of total glioma cells [24], we found that recruited macrophages (GFP*^+^*/CD11b^+^: 57.97% ± 2.96%) were almost two times as common as resident microglia (GFP^−^/CD11b^+^: 39.27% ± 1.84%) (Figure 4Db). *Sr-a1^+/+^* BMDMs accounted for approximately 70% of all *Sr-a1^+/+^* bone marrow cells (Supplementary Figure S5). These data indicate that BMDM may be the main cell to express SR-A1 in the glioma microenvironment.

FACS analysis showed that the number of BMDMs (F4/80^+^/CD45^high^, *Sr-a1*^+/+^ glioma: 57.3% ± 5.71%, *Sr-a1*^−/−^ glioma: 74.0% ± 4.05%) but not microglia (F4/80^+^/CD45^low^, *Sr-a1*^+/+^ glioma: 42.1% ± 5.63%, *Sr-a1*^−/−^ glioma: 25.6% ± 4.25%) was significantly increased in *Sr-a1*^−/−^ gliomas compared with *Sr-a1*^+/+^ gliomas (Figure 4E), suggesting that SR-A1 deficiency promoted BMDM infiltration into tumors. Again, there were more M2-like (F4/80^+^/CD206^+^) and less M1-like (F4/80^+^/CD11c^+^) bone marrow-derived TAMs (CD45^high^) in *Sr-a1*^−/−^ glioma, but there was no significant difference in microglia (CD45^low^) (M1 BMDM: *Sr-a1*^+/+^ glioma: 41.25% ± 4.68%, *Sr-a1*^−/−^ glioma: 16.7% ± 2.33%; M2 BMDM: *Sr-a1*^+/+^ glioma: 36.3% ± 7.55%, *Sr-a1*^−/−^ glioma: 60.88% ± 2.20%) (Figure 4F). These results clearly indicate that SR-A1 deletion led to increased bone marrow-derived M2-like TAM polarization in the murine glioma.

### SR-A1 inhibits tumor growth, invasion and angiogenesis through shifting M2-like BMDM polarization

**Figure 5:**
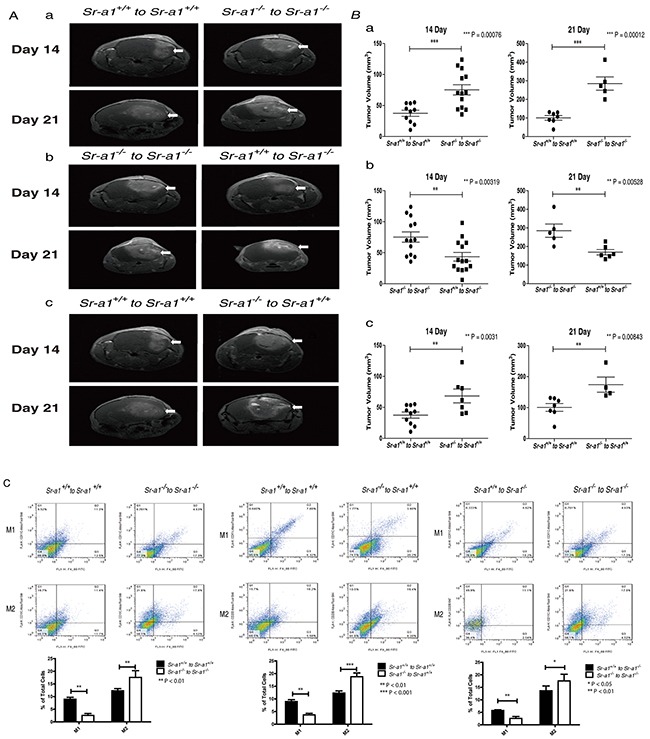
SR-A1 in BMDMs inhibits tumor growth and angiogenesis in the murine brain Contrast-enhanced T1-weighted magnetic resonance images of the bone marrow transplanted mouse brain on days 14 and 21 after intracranial implantation of GL261 murine glioma cells **A.** Quantitative analysis of tumor volume measured by magnetic resonance images. (n = 6; ***P<0.001) **B.** Flow cytometry analysis of macrophage polarization in the orthotopic glioma after bone marrow transplantation C. *Sr-a1*^+/+^ to *Sr-a1*^+/+^ comparing with *Sr-a1*^−/−^ to *Sr-a1*^−/−^ **Ca.**; *Sr-a1*^+/+^ to *Sr-a1*^+/+^ comparing with *Sr-a1*^−/−^ to *Sr-a1*^+/+^ **Cb.**; *Sr-a1*^+/+^ to *Sr-a1*^−/−^ comparing with *Sr-a1*^−/−^ to *Sr-a1*^−/−^ **Cc.** F4/80^+^/CD11c^+^ dot plots (right upper quadrant) represent M1-like microglia/macrophages. F4/80^+^/CD206^+^ dot plots (right upper quadrant) represent M2-like microglia/macrophages (n = 6; **P<0.01).

To determine if M2-like BMDM infiltration and polarization could be reversed by restoring SR-A1 function in BMDMs, we transplanted bone marrow cells from donors of different genetic backgrounds into lethally irradiated recipient mice and then orthotopically inoculated murine glioma cells after 4 weeks of recovery. Transplantation itself did not change orthotopic glioma phenotypes in mice (tumor volumes 21 days post inoculation: *Sr-a1^+/+^* to *Sr-a1^+/+^*: 42.9 ± 14.8 mm^3^; *Sr-a1^−/−^* to *Sr-a1^−/−^*: 81.9 ± 27.5 mm^3^) (Figure 5Aa & 5Ba). However, transplantation of *Sr-a1*^−/−^ bone marrow into *Sr-a1*^+/+^ mice accelerated glioma progression compared with *Sr-a1*^+/+^ to *Sr-a1*^+/+^ transplantation (*Sr-a1^−/−^* to *Sr-a1^+/+^*: 68.9 ± 42.2 mm^3^). In contrast, restoration of SR-A1 expression in BMDMs markedly inhibited tumor growth (*Sr-a1^+/+^* to *Sr-a1^−/−^*: 44.7 ± 24.7 mm^3^). These changes were evident 14 days and 21 days post inoculation (Figure 5Ab, 5Ac, 5Bb & 5Bc). Consistent with MRI results, PCNA, VEGF and CD31 immunohistochemical staining revealed that *Sr-a1^+/+^* to *Sr-a1^−/−^* transplantation led to reduced proliferation and angiogenesis in gliomas (Supplementary Figure S6). Transplantation itself did not influence SR-A1-related BMDM polarization as measured by a FACS analysis (Figure 5Ca). However, the M2-like BMDM phenotype was observed more frequently in *Sr-a1*^+/+^ mice receiving *Sr-a1*^−/−^ bone marrow (Figure 5Cb). Restoration of SR-A1 expression by bone marrow transplantation in *Sr-a1*^−/−^ mice led to decreased M2-like (CD206*^+^*) and increased M1-like (CD11c*^+^*) macrophage polarization (Figure 5Cc).

**Figure 6:**
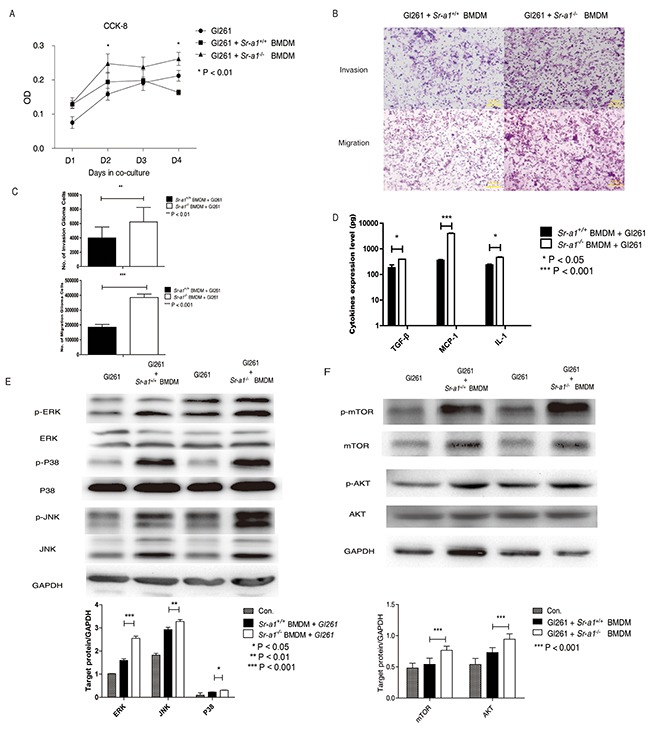
SR-A1 in BMDMs promotes glioma cells growth and invasion *in vitro* Measurements of GL261 cell proliferation **A.** 5×10^3^ GL261 cells and 1×10^5^ murine BMDMs were seeded to bottom and top plates, respectively. CCK-8 activity in GL261 cells was measured as a cell proliferation index (n = 3; **P<0.01). Representative hemosan staining of the penetrated cells in transwell invasion and migration assays **B.** 1×10^4^ GL261 cells were co-cultured with 1×10^5^ murine BMDMs for 18 h. Quantitative analysis of the penetrated cells in transwell invasion and migration assay (n = 6; **P<0.01; ***P<0.001) **C.** ELISA analysis of MCP-1, IL-1 and TGF-β expression in Gl261 cells co-cultured with 1×10^5^ murine BMDMs (n = 6: *P<0.05; **P<0.01) **D.** Representative and quantitative western blot analysis of the MAPK **E.** and PI3K **F.** signaling pathways in GL261 cells co-cultured with 1×10^5^ murine BMDMs for 6 h. Change in target protein level was compared with GAPDH (**P<0.01; ***P<0.001).

We found that glioma cells (GL261) co-cultured with *Sr-a1*^−/−^ BMDMs had a higher proliferation rate than those cultured with *Sr-a1*^+/+^ BMDMs (Figure 6A). GL261 cells also exhibited enhanced migration and invasion in the presence of *Sr-a1*^−/−^ BMDMs compared with *Sr-a1*^+/+^ BMDMs (Figure 6B & 6C). *Sr-a1*^−/−^ BMDMs produced more pro-tumorigenic factors such as IL-1, TGF-β and MCP-1 than *Sr-a1*^+/+^ BMDMs as measured by ELISA (Figure 6D). Western blotting revealed increased activation of MAPK (ERK1/2, p38 and JNK) and PI3K (mTOR and AKT) signaling in GL261 cells co-cultured with *Sr-a1*^−/−^ BMDMs as compared to *Sr-a1*^+/+^ BMDMs (Figure 6E & 6F).

**Figure 7:**
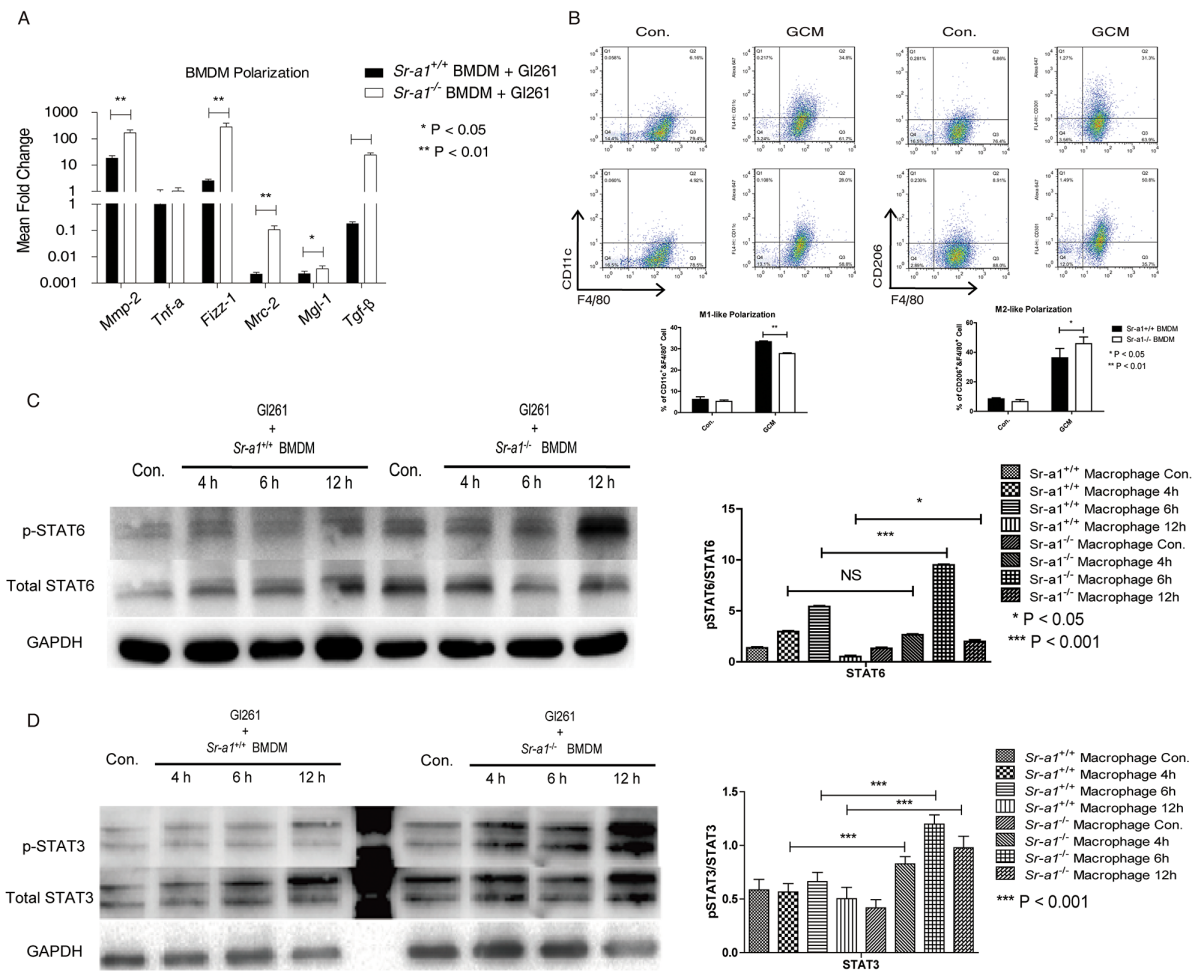
Effects of SR-A1 on BMDM polarization *in vitro* qPCR measurements of macrophage polarization signature genes in murine BMDMs (1×10^5^) co-cultured with equal numbers of GL261 cells for 12 h (n = 6; *P<0.05; **P<0.01; ***P<0.001) **A.** Flow cytometry analysis of BMDM polarization co-cultured with equal numbers of Gl261 cells **B.** F4/80^+^/CD11c^+^ dot plots represent M1-like BMDMs. F4/8^+^/CD301^+^ dot plots represent M2-like BMDMs (n = 6; *P<0.05; **P<0.01). Representative STAT6 **C.** and STAT 3 **D.** western blots in murine BMDMs (1×10^6^) with equal numbers of GL261 cells in the transwell co-culture system for 4 or 6 h. Changes in target protein levels were compared with total STAT6 or STAT3 (*Sr-a1*^+/+^group and *Sr-a1*^−/−^group was separated by irrelevant protein sample).

We also evaluated the influence of SR-A1 on macrophage polarization *in vitro*. SR-A1 deficiency resulted in up-regulation of M2-like markers (*Mmp-2, Tgf-β, Mrc-2, Mgl-1, Fizz-1*), but no significant changes in M1-like markers (*Tnf-a*) in the presence of GL261 cells (Figure 7A). These results were corroborated by FACS analysis of the macrophage subtypes (Figure 7B). These data support the hypothesis that BMDM-specific SR-A1 might inhibit glioma progression by preventing M2-like polarization.

Finally, we explored the potential signaling cascades through which SR-A1 may regulate M2-like BMDM polarization. We found that co-culture with glioma cells induced STAT3 and STAT6 phosphorylation, two important signaling molecules controlling M2-like macrophage differentiation in BMDMs [25]. SR-A1 deletion in BMDMs increased this effect (Figure 7C & 7D), suggesting that STAT3 and STAT6 signaling contribute to SR-A1 mediated BMDM polarization in the glioma microenvironment.

### HSP70 acts as an endogenous ligand for SR-A1 in glioma

**Figure 8:**
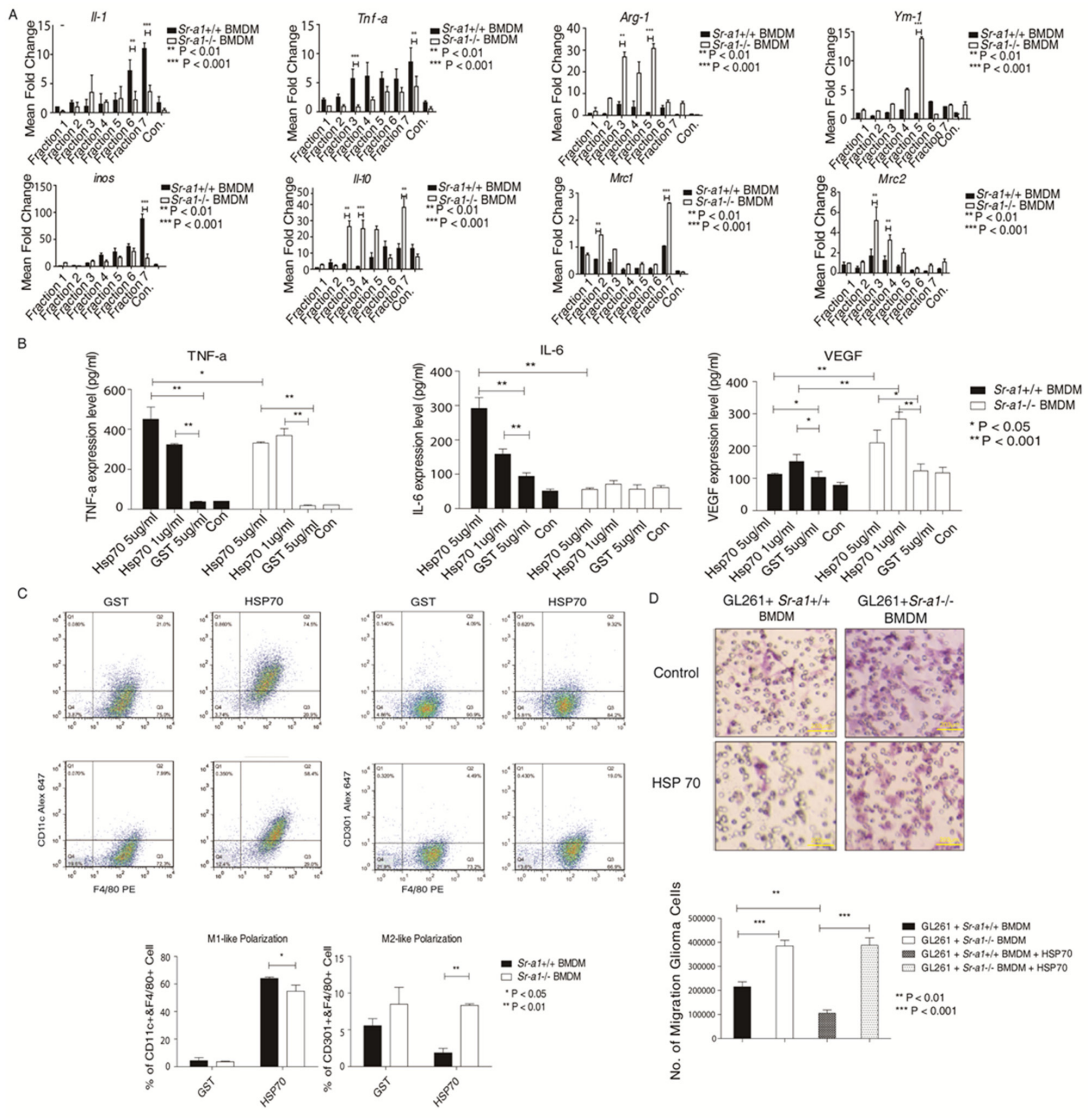
HSP70 inhibits glioma progression by activating SR-A1 in BMDMs qPCR measurements of cytokines in BMDMs induced by murine orthotopic glioma fractions (n = 6; *P<0.05; **P<0.01; ***P<0.001) **A.** ELISA for cytokines in BMDMs induced by HSP family **B.** BMDMs (1×10^5^) were treated with HSP70 (5 μg/ml), HSP40 (5 μg/ml), HSP60 (5 μg/ml) or GST (5 μg/ml) for 12 h (n = 6; *P<0.05; **P<0.01). Flow cytometry analysis of murine BMDMs pre-treated with HSP70 (5 μg/ml) **C.** F4/80+/CD11c^+^ dot plots represent M1-like macrophages. F4/80^+^/CD206^+^ dot plots represent M2-like macrophages (n = 6; *P<0.05; **P<0.01). Representative hemoson staining of the penetrated cells in transwell migration assay **D.** GL261 cells (1×10^4^) were co-cultured with murine BMDMs (1×10^5^) pre-treated with HSP70 (5 μg/ml) for 18 h (n = 6; ***P<0.001).

To identify putative endogenous SR-A1 ligand(s), we incubated mouse glioma lysates containing potential SR-A1 ligands with *Sr-a1*^+/+^ or *Sr-a1*^−/−^ BMDMs. Macrophage cytokine levels were measured to assess SR-A1 activation. qPCR results showed that treatment with fraction 3 (40% sucrose) or fraction 7 (30% sucrose) elevated M1-like cytokine production and suppressed that of M2-like cytokines in *Sr-a1*^+/+^ BMDMs compared with *Sr-a1*^−/−^ BMDMs (Figure 8A). The protein components of these two fractions were identified by SDS-PAGE coupled with liquid chromatography–mass spectrometry (LC-MS). Heat shock protein 70 (HSP70), which has been reported as a ligand for SR-A1, was detected among 47 identified proteins [26]. HSP70 expression in glioma was also confirmed by IHC analysis and western blot (Supplementary Figure S7).

To validate the role of HSP70/SR-A1 in the glioma microenvironment, we generated GST-fused recombinant HSP70, HSP60 and HSP40. qPCR and ELISA results indicated that treatment with GST-HSP70 elicited a strong pro-inflammatory response typical of M1-like macrophage activation only in the presence of SR-A1. No significant response was detected in GST alone (Figure 8B). Only some inflammatory cytokines, such as IL-1 and TNF-α, were up-regulated after treatment with HSP40 or HSP60 (Supplementary Figure S8). FACS analysis showed that HSP70 stimulated M1-like cell polarization more in *Sr-a1*^+/+^ BMDMs than in *Sr-a1*^−/−^ BMDMs, and promoted M2-like differentiation more in *Sr-a1*^−/−^ BMDMs (Figure 8C). Co-culture experiments revealed that HSP70 pretreatment inhibited GL261 cell migration in the presence of *Sr-a1*^+/+^ BMDMs, but not *Sr-a1*^−/−^ BMDMs (Figure 8D). IHC analysis and western blotting showed HSP70 expression in glioma tissues, but there was no significant difference in expression between *Sr-a1*^+/+^ and *Sr-a1*^−/−^ gliomas (Supplementary Figure S7). Finally, HSP70 treatment directly inhibited STAT3 and STAT6 phosphorylation in *Sr-a1*^+/+^ BMDMs, but had only a minor effect in *Sr-a1*^−/−^ BMDMs, confirming that the described HSP70 signaling effects require SR-A (Supplementary Figure S9). Thus, HSP70 might induce SR-A1 mediated M1-like macrophage polarization to suppress glioma progression.

## DISCUSSION

Of the multiple unique stromal cell types commonly found in solid tumors, TAMs are essential in fostering tumor progression [27, 28]. TAMs function in angiogenesis and produce soluble mediators to support tumor cell proliferation, survival and invasion [24]. Mechanisms of TAM communication with the tumor microenvironment remain poorly understood. This study suggests that HSP70/SR-A1-mediated signaling connects TAM polarization to glioma growth, invasion and angiogenesis.

TAM infiltration is correlated with enhanced glioma malignancy and poor prognosis [29-31], and approximately one third of all cells in glioma biopsies are labeled by macrophage markers [32, 33]. Macrophages and microglia are especially numerous in high-grade tumors [34], and ramified microglia (with preserved longer, perpendicularly branching cell processes) are abundant in gemistocytic astrocytomas [35]. A positive correlation was found between the number of CR3 (CD11b)-positive macrophages/microglia in gliomas and tumor proliferation rate [32]. Another striking feature, especially of high-grade gliomas, is the large number of immune cells (microglia and macrophages) that accumulate in the tumor mass (tumor-associated myeloid cells; TAMs). In GBM, TAMs can constitute up to 30% of the tumor mass [36] and, consistent with our observation that SR-A1 expression increased with glioma grade, are reportedly less abundant in lower grade gliomas. However, improved survival and reduced tumor recurrence were observed in patients with higher SR-A1 levels. We suggest that increased SR-A1 expression might be caused by TAM infiltration. Our studies showed increased SR-A1^+^/CD11b^+^ and decreased SR-A1^high^ macrophages during glioma development and progression in both human and murine gliomas. These findings indicate that SR-A1 may serve not only as a marker but also as a negative regulator of TAMs during glioma progression. SR-A1 may serve as a tumor suppressor during glioma progression, but might be suppressed by the glioma microenvironment, leading to its down-regulation in BMDMs co-cultured with glioma cells.

The origin of TAMs is heavily debated. TAMs may be derived from circulating monocytes attracted to the tumor tissue. Alternatively, TAMs can be converted from resident microglia under the instruction of the tumorigenic microenvironment [37]. As gliomas progress, abundant BMDMs are recruited to the tumor tissue and become the dominant macrophages possessing hypertrophic (M2-like) characteristics [38]. Our observations (Figure 4) indicate that BMDMs are a primary source of SR-A1 in the glioma microenvironment. In addition, bone marrow transplantation experiments (Figure 5) confirm that SR-A1 in BMDMs is largely, if not completely, accountable for its anti-tumorigenic activities. SR-A1 expression in tumor tissue TAMs, mostly recruited macrophages, correlates inversely with glioma malignancy in humans (Figure 1). This is confirmed by the observation that SR-A1 deficiency increases hypoxia (Figure 3), which is consistent with previous reports that local hypoxia supports angiogenesis and inflammation and contributes to tumor metastasis and progression [39].

The dynamic nature of TAMs during tumor progression is influenced in part by local concentrations of cytokines and chemokines, as well as varied interactions of TAMs with normal and malignant cells. Our study demonstrates for the first time that SR-A1 could inhibit TAM differentiation toward an M2-like phenotype by suppressing STAT3 and STAT6 signaling in murine gliomas. SR-A1 deletion increased the observed frequency of the TAM M2 subtype. Polarized TAMs influence tumor progression by releasing multiple cytokines, such as VEGF and MMP-9, to sustain angiogenesis and proliferation [40]. In agreement with our previous studies in lung cancer [16], we found that SR-A1 deletion was associated with overproduction of MMP-9, VEGF and other tumorigenic and angiogenic factors (Figure 3 & Supplementary Figure S3)

The interplay between tumor cells and stromal cells determines the glioma tissue phenotype [41]. Many glioma-related genes are expressed comparably in both tumor cells and stromal cells, although they usually function differently in different cell types. For example, while TLRs in tumor cells facilitate evasion of immune surveillance and thus promote tumor growth, they polarize macrophages to an anti-tumor M1-like phenotype [28, 42]. This dichotomy makes targeting these molecules for cancer treatment challenging [43]. SR-A1 is expressed only in TAMs, making it an ideal protein for stromal cell-specific tumor therapy. Our data highlight a role for SR-A1 in the regulation of a dynamic interplay between tumor cells and stromal cells. On one hand, tumor cells promote BMDM recruitment into glioma tissues (Figure 4E & 4F). SR-A1 deficiency seems to amplify this effect, likely as a consequence of enhanced p38 phosphorylation [44]. Reciprocally, infiltrated BMDMs induce tumor cells to alter their migratory and invasive behaviors. SR-A1 deficiency appears to enhance this effect by activating STAT3/STAT6 signaling and routing macrophages toward an M2-like phenotype. This type of cell-cell interaction appears to be context-dependent as we observed that SR-A1 influenced macrophage infiltration at the tumor center but not around the edges (Figure 4H). SR-A1 may serve a key gatekeeper, blocking communication between tumor cells and stromal cells and preventing glioma deterioration.

SR-A1 appeared to influence macrophage proliferation, infiltration and differentiation simultaneously. Robbins, *et al.* reported that SR-A1 is necessary for promoting resident macrophage proliferation in mouse atherosclerotic lesions, while others have shown the SR-A1 deficiency enhances macrophage recruitment in different diseases [45-48]. We recently showed that SR-A1 deficiency could enhance RAGE expression and function [49], and others showed that RAGE expression in TAMs could enhance glioma progression by promoting angiogenesis [50]. Thus, SR-A1-dependent macrophage infiltration may alter the local microenvironment, which in turn may impact cell proliferation and/or death, shaping a pro-M1 or pro-M2 setting to promote differentiation.

Finally, our results demonstrate that HSP70 could be an endogenous ligand that activates SR-A1-dependent anti-tumorigenic pathways in gliomas. HSPs are over-expressed in a wide range of human cancers and are implicated in tumor cell proliferation, differentiation, invasion, metastasis, death and recognition by the immune system [51, 52]. Our results show that HSP70, but not HSP40 or 60, is specifically involved in glioma progression, although its expression does not appear informative for diagnostic purposes. Elevated HSP70 expression correlates with poor prognosis in breast, endometrial, uterine, cervical and bladder carcinomas [53]. However, its expression predicts improved response to chemotherapy in osteosarcomas and glioma [53]. This likely reflects the complex and unique nature of the tumor microenvironment in different tissues and organs.

Although HSP70 by itself is sufficient to induce an M1-like phenotype in macrophages *in vitro*, its precise role in glioma pathogenesis is unclear. HSP70-induced phenotypic alterations of SR-A1-null macrophages suggest that HSP70 has SR-A1 independent functions yet to be determined. Additionally, Neyen, *et al.* identified several SR-A1 ligands that do not completely overlap with those found in the present study in models of ovarian and pancreatic cancer [54]. This apparent discrepancy highlights the importance of the tumor microenvironment in SR-A1 activity.

In summary, our findings demonstrate a relationship between TAMs, SR-A1 expression and glioma growth and provide new insights into the pathogenic role of TAMs in glioma. The HSP70/SR-A1 pathway may inhibit STAT3/6 signaling in TAMs to slow glioma progression. Our present study suggests that inhibiting macrophage recruitment and influencing macrophage polarization (away from an M2-like phenotype) with SR-A1 ligands such as HSP70 could reduce glioma development and progression.

## MATERIALS AND METHODS

### Samples

We evaluated specimens resected from primary glioma patients between 2010 and 2014 at the Brain Hospital Affiliated with Nanjing Medical University (Table 2). Informed written consent was obtained from all patients under protocols approved by the Institutional Review Board of Nanjing Medical University. Two pathologists provided histological diagnoses according to the revised 2008 WHO classification [55]. Patients were divided into SR-A1 high and low expression groups according to median SR-A1 expression level.

**Table 2:** Characteristics of human glioma samples used in this study

	Grade II	Grade III	Grade IV
No. of cases	48	46	42
Gender			
Male	20	21	28
Female	28	25	14

### Animals, tumor implantation and bone marrow transplantation

All animal protocols were reviewed and approved by the intramural Ethics Committee on Humane Treatment of Experimental Animals. SR-A1^+/+^ and GFP^+^ C57BL6 mice (6–8 weeks old) were obtained from the animal colony of Nanjing Medical University. SR-A1^−/−^ C57BL6 mice were obtained from the Jackson Laboratory (Stock number: 006096) and generated in Nanjing Medical University. Mice were bred and maintained under pathogen-free conditions with a 12:12-h light: dark cycle and regular chow diet and water.

### Magnetic resonance imaging and evaluation of tumor volume

On days 14 and 21 after tumor implantation, animals were anesthetized and glioma images were acquired as described previously [56].

### Histopathology and immunohistochemistry

Tumors were fixed in 4% paraformaldehyde overnight. Samples used for paraffin sections were transferred to 10% neutral-buffered formalin and embedded in paraffin wax. Sections (4 μm) were prepared, stained with hematoxylin and eosin (H&E) and examined microscopically. Tumors used for cryosections were washed with phosphate-buffered saline (PBS) after fixation and placed in 30% sucrose at 4°C before being transferred into optimal cutting temperature-embedding compound.

### Bone marrow derived macrophage isolation

Bone marrow cells were harvested and cultured as described previously [57].

### Immunohistochemical analysis

Primary antibodies used were SR-A1 (1:200, BMA Biomedical, Swiss), F4/80 (1:200, AbD Serotec, UK), CD16/32 (1:200, BD Biosciences Pharmingen, USA), CD206 (1:100, AbD Serotec), PCNA (1:100, AbD Serotec), CD34 (1:100, AbD Serotec), CD31 (1:100, AbD Serotec) or MMP9 (1:100, Abcam, USA). Secondary antibodies (1:500, BOSTER Bio-technology, China) and a detailed protocol were described previously [58].

### Flow cytometry

Whole brain hemispheres were broken into single cells using a syringe plunger and filtrated through a 70-μm cell strainer. Erythrocytes were lysed using the erythrolyse solution (Sigma-Aldrich, USA), and the remaining cells were resuspended in PBS (10^6^/100 μL PBS). Before staining, cells were permeabilized using the Leucoperm kit (Serotec). Cells were then incubated with the primary antibody in the dark for 45 min at room temperature, washed with 5 mL PBS and resuspended in 400 μL 2% paraformaldehyde in PBS. Fluorescence-labeled cells were counted using the FACS Calibur cytometer connected to CellQuest software (BD Biosciences).

Primary antibodies used were SR-A1 (1:200, BMA Biomedical), FITC-labeled antibody against F4/80 (1:200, AbD Serotec), APC-labeled antibody against CD11b (1:200, AbD Serotec), PE-labeled antibody against CD45 (1:200, BD Biosciences Pharmingen), PE-labeled antibody against SR-A1 (eBiosciences, USA), PE-labeled antibody against VCAM-1 (eBiosciences), Alexa Flour 647-labeled antibody against CD11c (1:200, eBioscience), Alexa Flour 647-labeled antibody against CCR2^+^ (eBioscience), Alexa Flour 647-labeled antibody against CD206 (1:200, AbD) and anti-human CD206 (MMR) APC (1:200, eBioscience). The gating scheme for all experiments included first using forward and side scatter along with Fixable Viability Dye (eBioscience) to identify live singlet lymphocytes. Subsequent gating parameters are described in the figure legends.

### Total RNA purification and real-time PCR analysis

Total RNA was isolated from 100 mg of tumor tissue using the RNeasy extraction kit (Takara, Japan). RNA yield and purity were determined by spectrophotometer. cDNA synthesis was done with 500 ng total RNA using the cDNA synthesis kit (Takara). qRT-PCR was done using SYBR Green ER qPCR Super mix (Roche, Swiss) and an AB7500 system (Seegene, USA). Primers were designed by Takara. Expression of each target gene was normalized to that of the control gene, β-actin or GAPDH.

### Bone marrow-derived macrophage isolation

BMDMs were prepared as previously described [59]. BMDMs were maintained in RPMI 1640 (Invitrogen) supplemented with 10% fetal bovine serum (HyClone), 20% L929 cell supernatant, 0.5% mercaptoethanol and 1% penicillin-streptomycin at 37°C with 5% CO_2_ for 5 days before experimentation.

### Western blotting

Primary antibodies used in western blotting in this study were specific for SR-A1 (Abcam), VEGF (Abcam), β-actin (Santa Cruse), HSP70 (R&D Systems, USA), STAT3 (Cell signaling, USA), STAT6 (Cell signaling), p-mTOR (Cell signaling), mTOR (Cell signaling), p-AKT (Cell signaling), AKT (Cell signaling), p-ERK (Cell signaling), ERK (Cell signaling), p-JNK (Cell signaling), JNK (Cell signaling), p-P38 (Cell signaling), P38 (Cell signaling) and GAPDH (Cell Signaling). Detailed protocol was described previously [60].

### Macrophage and glioma cell co-culture system

GL261 cells were plated with murine primary BMDMs. In brief, primary *Sr-a1*^+/+^ and *Sr-a1*^−/−^ macrophages were isolated and re-suspended at a density of 5×10^4^ cells/ml in DMEM with 1% FBS. After 5 days, medium was removed and 2×10^4^ GL261 cells were seeded on top of the BMDMs in DMEM with 10% FBS, and cells were co-cultured for 4, 6 and 12 h. As controls, primary macrophages were switched to the same medium without GL261 cells. Macrophage RNA was isolated and quantitatively assessed via qRT-PCR. The experiments were repeated three times with duplicate samples per group. Segregated macrophage-glioma co-cultures were prepared as follows: 5×10^4^ *Sr-a1*^−/−^ or *Sr-a1*^+/+^ macrophages were seeded on 0.4 μm inserts (Millicell, USA) in DMEM with 1% FBS. After 5 days, inserts were moved to 24-well plates containing 5,000 GL261 cells/well in DMEM with 10% FBS. Empty inserts with the same medium were used as controls. GL261 proliferation was measured every other day. Experiments were repeated three times with duplicate samples per group.

### Cell proliferation assay

The CCK-8 kit (Beyotime, China) was used to measurement cell proliferation. In brief, equal numbers of GL261 cells were seeded in 96-well or 24-well plates (co-culture) with respective treatments (duplicate samples for each treatment). At each time point, the medium was changed to include a 1:10 dilution of CCK-8. After 2 h at 37°C, absorbance was read at 450 nm. Absorbance values at each time point were normalized using those at day 0.

### Preparation and LC-MS analysis of brain lysate

Mice were perfused with PBS transcardially. The brain glioma was removed, homogenized with RPMI-1640 and centrifuged at 15,000 rpm for 5 min. The supernatant was made up to 1 ml with RPMI-1640 and used as the brain lysate. For digestion assays, brain lysate was incubated with pronase (1–10 U/ml, Roche, USA) or DNase I (50 μg/ml, Roche) at 37°C for 1 h. For the sucrose density gradient centrifugation and LC-MS analysis, the brain lysate was ultracentrifuged at 47,000 rpm for 1 h. The supernatant was applied to DEAE sepharose fast flow columns (GE Healthcare, USA), and the flow-through was condensed by ultrafiltration using an Amicon Ultra-4 centrifugal filter unit with Ultracel-10 membrane (Millipore, USA). Four hundred microliters of condensed solution was layered on a 1-ml 10–40% (w/w) linear sucrose gradient in PBS and centrifuged at 40,000 rpm for 12 h. Sucrose was depleted by ultrafiltration from each of the sucrose gradient fractions. We added the sucrose-gradient fractions to cultures of SR-A1^+/+^ and SR-A1^−/−^ macrophages to examine induction of macrophage polarization cytokine expression. LC-MS analysis of sucrose gradient fractions 1–7 was performed after trypsinization using a Qstar-XL mass spectrometer (Applied Biosystems, USA).

### Generation of recombinant protein

HSP40, 60 and 70 recombinant proteins with GST tags were kindly provided by Dr. Su Chuan from Nanjing Medical University. Murine IL-4 (100 ng/ml), IL-13 (100 ng/ml) and LPS (50 ng/ml) recombinant proteins were acquired from Sigma-Aldrich. Recombinant proteins were co-cultured with SR-A1^+/+^ and SR-A1^−/−^ macrophages and recombinant GST protein was used as a negative control for cytokine induction in macrophages.

### Assays

TGF-β, MCP-1 and IL-10 levels in tumor homogenates and plasma were determined using mouse TGF-β, mouse MCP-1 and mouse IL-10 ELISA Kits according to the manufacturer's protocols (Excell, China). Plates were read on a Bio-Rad (USA) Model 680 microplate reader at 415 nm. Protein antibody array data were provided by Ray Biotech Inc, China.

### Magnetic resonance imaging and evaluation of tumor volume

On days 14 and 21 after tumor implantation, animals were anesthetized with 2% isoflurane (Abbott GmbH & Co. KG, Germany) in a 20/80 oxygen/air gas stream to acquire glioma images as described below. A Bruker (Germany) Pharma Scan 7.0T MR imager (300.51MHz for 1 h, 300 mT/m gradient system) was used to acquire images in T1-weighted [TR = 451.4 ms, TE = 5.5 ms, 1 acquisition (NEX 1 averages), TA = 1 min 55 s, field of view = 2.7×2.7 cm, matrix = 256×256, slice thickness = 1.00 mm, voxelsize = 10.5×10.5×1 mm^3^] and T2-weighted [TR = 3081.0 ms, TE = 72.0 ms, 1 acquisition (NEX 1 averages), TA = 6 min 34 s, field of view = 2.5×2.5 cm, matrix = 512×128, slice thickness = 1.5 mm, voxelsize = 4.9×19.5×1.5 mm^3^] images using spin echo sequences. Coronal serial T2-weighted scans were obtained before contrast medium injection. Mice received 80 μL of gadolinium DTPA (Magnevist, Bayer-Schering Pharma, Germany) intraperitoneally and T1-weighted scans were taken for 10 min.

Tumor size was calculated in T1-weighted image sets obtained 10 min after contrast injection. Slices showing enhanced areas were analyzed by measuring the area of a region of interest around the section of enhancement. Volume was calculated by multiplying by the slice thickness. This procedure was repeated for all slices showing enhancement and the areas were summed to determine a total volume.

### Statistical analysis

All experiments were performed at least three times. In Kaplan–Meier curves, survival differences were compared by log-rank analysis. All statistical analyses were performed using Statistical Package for Social Science (SPSS) 13.0 software. Statistical significance was assessed by Student's t test (unpaired two-tailed). P<0.05 (*), 0.01 (**) or 0.001 (***) was considered statistically significant.

## SUPPLEMENTARY MATERIALS FIGURES



## References

[1] Markovic DS, Vinnakota K, Chirasani S, Synowitz M, Raguet H, Stock K, Sliwa M, Lehmann S, Kalin R, van Rooijen N, Holmbeck K, Heppner FL, Kiwit J (2009). Gliomas induce and exploit microglial MT1-MMP expression for tumor expansion. Proceedings of the National Academy of Sciences of the United States of America.

[2] Louis DN, Ohgaki H, Wiestler OD, Cavenee WK, Burger PC, Jouvet A, Scheithauer BW, Kleihues P (2007). The 2007 WHO classification of tumours of the central nervous system. Acta neuropathologica.

[3] Kessenbrock K, Plaks V, Werb Z (2010). Matrix metalloproteinases: regulators of the tumor microenvironment. Cell.

[4] Bagley RG (2010). The tumor microenvironment. (New York: Springer).

[5] Rigoni A, Colombo MP, Pucillo C (2014). The Role of Mast Cells in Molding the Tumor Microenvironment. Cancer microenvironment.

[6] Pollard JW (2004). Tumour-educated macrophages promote tumour progression and metastasis. Nature reviews Cancer.

[7] Condeelis J, Pollard JW (2006). Macrophages: obligate partners for tumor cell migration, invasion, and metastasis. Cell.

[8] Gallina G, Dolcetti L, Serafini P, De Santo C, Marigo I, Colombo MP, Basso G, Brombacher F, Borrello I, Zanovello P, Bicciato S, Bronte V (2006). Tumors induce a subset of inflammatory monocytes with immunosuppressive activity on CD8+ T cells. The Journal of clinical investigation.

[9] Dieterich LC, Mellberg S, Langenkamp E, Zhang L, Zieba A, Salomaki H, Teichert M, Huang H, Edqvist PH, Kraus T, Augustin HG, Olofsson T, Larsson E (2012). Transcriptional profiling of human glioblastoma vessels indicates a key role of VEGF-A and TGFbeta2 in vascular abnormalization. The Journal of pathology.

[10] Franklin RA, Liao W, Sarkar A, Kim MV, Bivona MR, Liu K, Pamer EG, Li MO (2014). The cellular and molecular origin of tumor-associated macrophages. Science.

[11] Rosas M, Davies LC, Giles PJ, Liao CT, Kharfan B, Stone TC, O'Donnell VB, Fraser DJ, Jones SA, Taylor PR (2014). The transcription factor Gata6 links tissue macrophage phenotype and proliferative renewal. Science.

[12] Tumor-associated macrophages are distinct from normal macrophages (2014). Cancer discovery.

[13] Tripodo C, Sangaletti S, Piccaluga PP, Prakash S, Franco G, Borrello I, Orazi A, Colombo MP, Pileri SA (2011). The bone marrow stroma in hematological neoplasms–a guilty bystander. Nature reviews Clinical oncology.

[14] Wang B, Liu H, Dong X, Wu S, Zeng H, Liu Z, Wan D, Dong W, He W, Chen X, Zheng L, Huang J, Lin T (2015). High CD204+ tumor-infiltrating macrophage density predicts a poor prognosis in patients with urothelial cell carcinoma of the bladder. Oncotarget.

[15] Komohara Y, Ohnishi K, Kuratsu J, Takeya M (2008). Possible involvement of the M2 anti-inflammatory macrophage phenotype in growth of human gliomas. The Journal of pathology.

[16] Ben J, Jin G, Zhang Y, Ma B, Bai H, Chen J, Zhang H, Gong Q, Zhou X, Zhang H, Qian L, Zhu X, Li X (2012). Class A scavenger receptor deficiency exacerbates lung tumorigenesis by cultivating a procarcinogenic microenvironment in humans and mice. American journal of respiratory and critical care medicine.

[17] Sangaletti S, Di Carlo E, Gariboldi S, Miotti S, Cappetti B, Parenza M, Rumio C, Brekken RA, Chiodoni C, Colombo MP (2008). Macrophage-derived SPARC bridges tumor cell-extracellular matrix interactions toward metastasis. Cancer research.

[18] Hu Y, Zhang H, Lu Y, Bai H, Xu Y, Zhu X, Zhou R, Ben J, Chen Q (2011). Class A scavenger receptor attenuates myocardial infarction-induced cardiomyocyte necrosis through suppressing M1 macrophage subset polarization. Basic research in cardiology.

[19] Xu Y, Qian L, Zong G, Ma K, Zhu X, Zhang H, Li N, Yang Q, Bai H, Ben J, Li X, Chen Q (2012). Class A scavenger receptor promotes cerebral ischemic injury by pivoting microglia/macrophage polarization. Neuroscience.

[20] Zhu X, Zong G, Zhu L, Jiang Y, Ma K, Zhang H, Zhang Y, Bai H, Yang Q, Ben J, Li X, Xu Y, Chen Q (2014). Deletion of class A scavenger receptor deteriorates obesity-induced insulin resistance in adipose tissue. Diabetes.

[21] Prabhudas M, Bowdish D, Drickamer K, Febbraio M, Herz J, Kobzik L, Krieger M, Loike J, Means TK, Moestrup SK, Post S, Sawamura T, Silverstein S, Wang XY, El Khoury J (2014). Standardizing scavenger receptor nomenclature. Journal of immunology.

[22] Pucci F, Venneri MA, Biziato D, Nonis A, Moi D, Sica A, Di Serio C, Naldini L, De Palma M (2009). A distinguishing gene signature shared by tumor-infiltrating Tie2-expressing monocytes, blood “resident” monocytes, and embryonic macrophages suggests common functions and developmental relationships. Blood.

[23] Hanisch UK, Kettenmann H (2007). Microglia: active sensor and versatile effector cells in the normal and pathologic brain. Nature neuroscience.

[24] Lewis CE, Pollard JW (2006). Distinct role of macrophages in different tumor microenvironments. Cancer research.

[25] Farren MR, Carlson LM, Netherby CS, Lindner I, Li PK, Gabrilovich DI, Abrams SI, Lee KP (2014). Tumor-induced STAT3 signaling in myeloid cells impairs dendritic cell generation by decreasing PKCbetaII abundance. Science signaling.

[26] Theriault JR, Adachi H, Calderwood SK (2006). Role of scavenger receptors in the binding and internalization of heat shock protein 70. Journal of immunology.

[27] Ruffell B, Affara NI, Coussens LM (2012). Differential macrophage programming in the tumor microenvironment. Trends in immunology.

[28] Huang B, Zhao J, Li H, He KL, Chen Y, Chen SH, Mayer L, Unkeless JC, Xiong H (2005). Toll-like receptors on tumor cells facilitate evasion of immune surveillance. Cancer research.

[29] Ding P, Wang W, Wang J, Yang Z, Xue L (2014). Expression of tumor-associated macrophage in progression of human glioma. Cell biochemistry and biophysics.

[30] Pyonteck SM, Akkari L, Schuhmacher AJ, Bowman RL, Sevenich L, Quail DF, Olson OC, Quick ML, Huse JT, Teijeiro V, Setty M, Leslie CS, Oei Y (2013). CSF-1R inhibition alters macrophage polarization and blocks glioma progression. Nature medicine.

[31] Komohara Y, Horlad H, Ohnishi K, Fujiwara Y, Bai B, Nakagawa T, Suzu S, Nakamura H, Kuratsu J, Takeya M (2012). Importance of direct macrophage-tumor cell interaction on progression of human glioma. Cancer science.

[32] Hirota S, Miyamoto M, Kasugai T, Kitamura Y, Morimura Y (1990). Crystalline light-chain deposition and amyloidosis in the thyroid gland and kidneys of a patient with myeloma. Archives of pathology & laboratory medicine.

[33] Roggendorf W, Strupp S, Paulus W (1996). Distribution and characterization of microglia/macrophages in human brain tumors. Acta neuropathologica.

[34] Morris CS, Esiri MM (1991). Immunocytochemical study of macrophages and microglial cells and extracellular matrix components in human CNS disease. 1. Gliomas. Journal of the neurological sciences.

[35] Wierzba-Bobrowicz T, Kuchna I, Matyja E (1994). Reaction of microglial cells in human astrocytomas (preliminary report). Folia neuropathologica.

[36] Zhou W, Ke SQ, Huang Z, Flavahan W, Fang X, Paul J, Wu L, Sloan AE, McLendon RE, Li X, Rich JN, Bao S (2015). Periostin secreted by glioblastoma stem cells recruits M2 tumour-associated macrophages and promotes malignant growth. Nature cell biology.

[37] Lawrence T, Natoli G (2011). Transcriptional regulation of macrophage polarization: enabling diversity with identity. Nature reviews Immunology.

[38] Qian BZ, Pollard JW (2010). Macrophage diversity enhances tumor progression and metastasis. Cell.

[39] Du R, Lu KV, Petritsch C, Liu P, Ganss R, Passegue E, Song H, Vandenberg S, Johnson RS, Werb Z, Bergers G (2008). HIF1alpha induces the recruitment of bone marrow-derived vascular modulatory cells to regulate tumor angiogenesis and invasion. Cancer cell.

[40] Cramer T, Yamanishi Y, Clausen BE, Forster I, Pawlinski R, Mackman N, Haase VH, Jaenisch R, Corr M, Nizet V, Firestein GS, Gerber HP, Ferrara N, Johnson RS (2003). HIF-1alpha is essential for myeloid cell-mediated inflammation. Cell.

[41] Hjelmeland AB, Lathia JD, Sathornsumetee S, Rich JN (2011). Twisted tango: brain tumor neurovascular interactions. Nature neuroscience.

[42] Sica A, Bronte V (2007). Altered macrophage differentiation and immune dysfunction in tumor development. The Journal of clinical investigation.

[43] Wilhelm SM, Adnane L, Newell P, Villanueva A, Llovet JM, Lynch M (2008). Preclinical overview of sorafenib, a multikinase inhibitor that targets both Raf and VEGF and PDGF receptor tyrosine kinase signaling. Molecular cancer therapeutics.

[44] Liu RY, Fan C, Liu G, Olashaw NE, Zuckerman KS (2000). Activation of p38 mitogen-activated protein kinase is required for tumor necrosis factor-alpha -supported proliferation of leukemia and lymphoma cell lines. The Journal of biological chemistry.

[45] Robbins CS, Hilgendorf I, Weber GF, Theurl I, Iwamoto Y, Figueiredo JL, Gorbatov R, Sukhova GK, Gerhardt LM, Smyth D, Zavitz CC, Shikatani EA, Parsons M (2013). Local proliferation dominates lesional macrophage accumulation in atherosclerosis. Nature medicine.

[46] Sun L, Wen JH, Sun HL, Shu XC, Hu F, Yin DC, Yang Q, Zeng YJ, Sun Y, Liu L (2012). Perindopril attenuates renal tubulointerstitium injury by inhibiting scavenger receptor A over-expression in diabetic rats. Journal of endocrinological investigation.

[47] Cotena A, Gordon S, Platt N (2004). The class A macrophage scavenger receptor attenuates CXC chemokine production and the early infiltration of neutrophils in sterile peritonitis. Journal of immunology.

[48] Usui HK, Shikata K, Sasaki M, Okada S, Matsuda M, Shikata Y, Ogawa D, Kido Y, Nagase R, Yozai K, Ohga S, Tone A, Wada J (2007). Macrophage scavenger receptor-a-deficient mice are resistant against diabetic nephropathy through amelioration of microinflammation. Diabetes.

[49] Ma K, Xu Y, Wang C, Li N, Li K, Zhang Y, Li X, Yang Q, Zhang H, Zhu X, Bai H, Ben J, Ding Q (2014). A crosstalk between class A scavenger receptor and receptor for advanced glycation end-products contributes to diabetic retinopathy. American journal of physiology Endocrinology and metabolism.

[50] Chen X, Zhang L, Zhang IY, Liang J, Wang H, Ouyang M, Wu S, Carvalho da Fonseca A, Weng L, Yamamoto Y, Yamamoto H, Natarajan R, Badie B (2014). RAGE Expression in Tumor-associated Macrophages Promotes Angiogenesis in Glioma. Cancer research.

[51] Ciocca DR, Calderwood SK (2005). Heat shock proteins in cancer: diagnostic, prognostic, predictive, and treatment implications. Cell stress & chaperones.

[52] Dodd K, Nance S, Quezada M, Janke L, Morrison JB, Williams RT, Beere HM (2014). Tumor-derived inducible heat-shock protein 70 (HSP70) is an essential component of anti-tumor immunity. Oncogene.

[53] Nylandsted J, Wick W, Hirt UA, Brand K, Rohde M, Leist M, Weller M, Jaattela M (2002). Eradication of glioblastoma, and breast and colon carcinoma xenografts by Hsp70 depletion. Cancer research.

[54] Neyen C, Pluddemann A, Mukhopadhyay S, Maniati E, Bossard M, Gordon S, Hagemann T (2013). Macrophage scavenger receptor a promotes tumor progression in murine models of ovarian and pancreatic cancer. Journal of immunology.

[55] Feiden S, Feiden W (2008). WHO classification of tumours of the CNS: revised edition of 2007 with critical comments on the typing und grading of common-type diffuse gliomas [Article in German]. Der Pathologe.

[56] Kerber M, Reiss Y, Wickersheim A, Jugold M, Kiessling F, Heil M, Tchaikovski V, Waltenberger J, Shibuya M, Plate KH, Machein MR (2008). Flt-1 signaling in macrophages promotes glioma growth in vivo. Cancer research.

[57] Stanley ER (1985). The macrophage colony-stimulating factor, CSF-1. Methods in enzymology.

[58] Xu Y, Qian L, Zong G, Ma K, Zhu X, Zhang H, Li N, Yang Q, Bai H, Ben J, Li X, Xu Y, Chen Q (2012). Class A scavenger receptor promotes cerebral ischemic injury by pivoting microglia/macrophage polarization. Neuroscience.

[59] Weischenfeldt J, Porse B (2008). Bone Marrow-Derived Macrophages (BMM): Isolation and Applications. CSH protocols.

[60] Zhu XD, Zhuang Y, Ben JJ, Qian LL, Huang HP, Bai H, Sha JH, He ZG, Chen Q (2011). Caveolae-dependent endocytosis is required for class A macrophage scavenger receptor-mediated apoptosis in macrophages. The Journal of biological chemistry.

