# Supplementary material for: Class A1 scavenger receptor modulates glioma progression by regulating M2-like tumor-associated macrophage polarization
Source: Oncotarget. 2016 Jun 29;7(31):50099–116. doi: 10.18632/oncotarget.10318 (PMC5226571; doi:10.18632/oncotarget.10318)
Supplement: Supplementary file 1 [file oncotarget-07-50099-s001.pdf]

Class A1 scavenger receptor modulates glioma progression by regulating M2-like tumor-associated macrophage polarization

SUPPLEMENTARY FIGURES

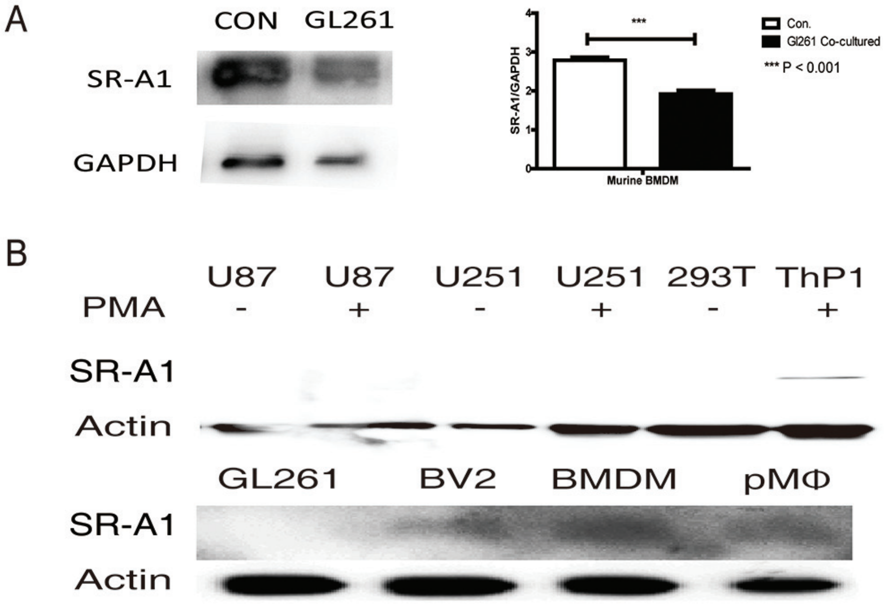

Supplementary Figure S1: Representative western blots of SR-A1 expression in murine BMDMs after co-culture with GL261 cells **A.** and in human glioma cell lines (U87, U251), a human macrophage cell line (ThP1), a murine glioma cell line (GL261), a murine macrophage cell line (RAW3.1), murine primary peritoneal macrophages and murine BMDMs **B.**

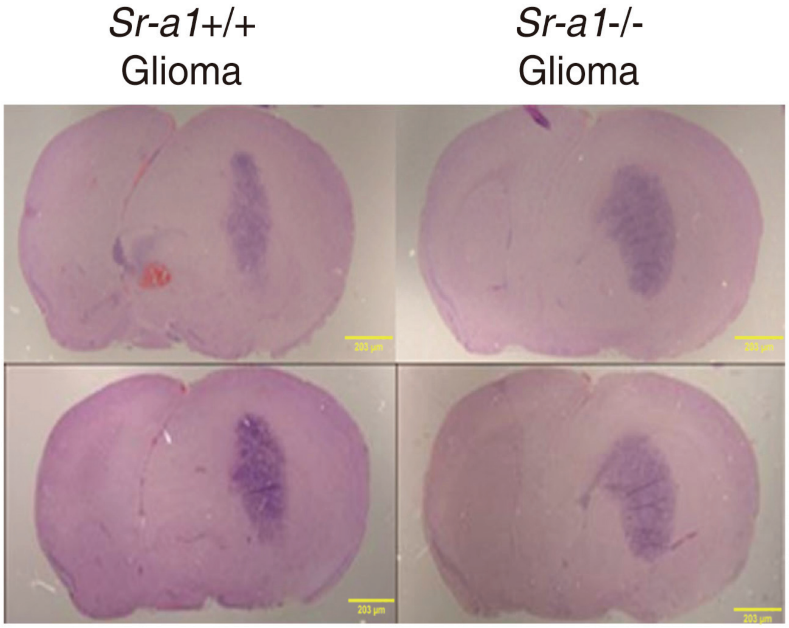

Supplementary Figure S2: Representative H&E staining of whole murine brain on day 21 post inoculation. n > 6.

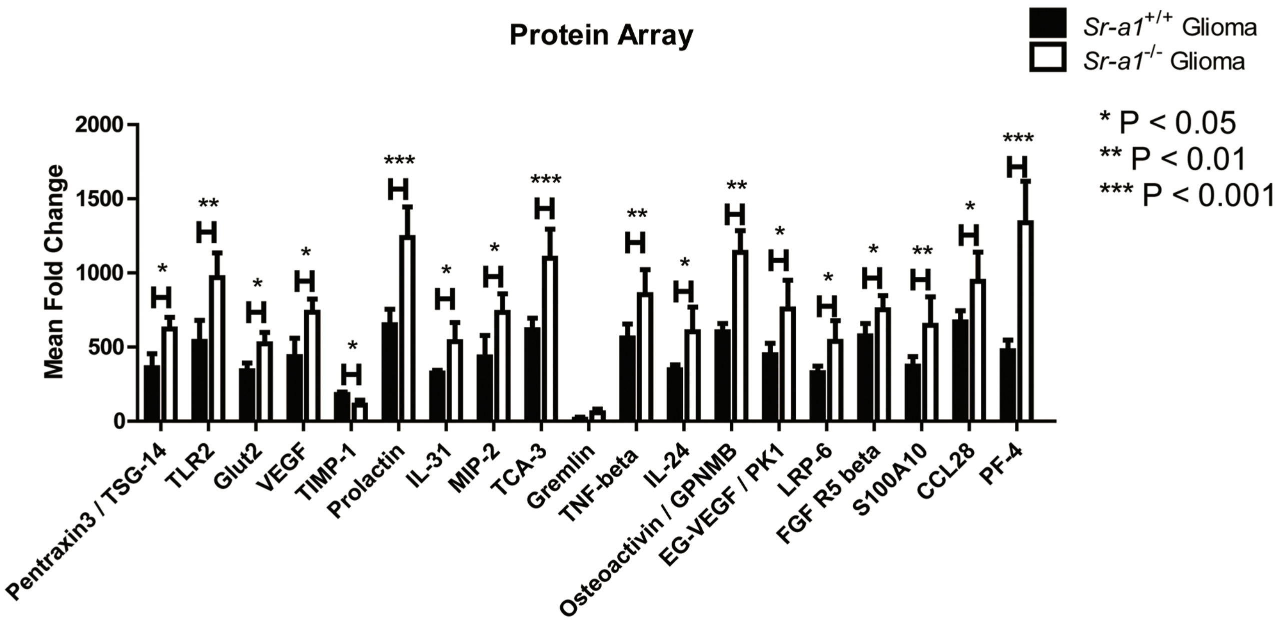

Supplementary Figure S3: Protein chip assay of murine orthotopic gliomas. n = 3; *P<0.05.

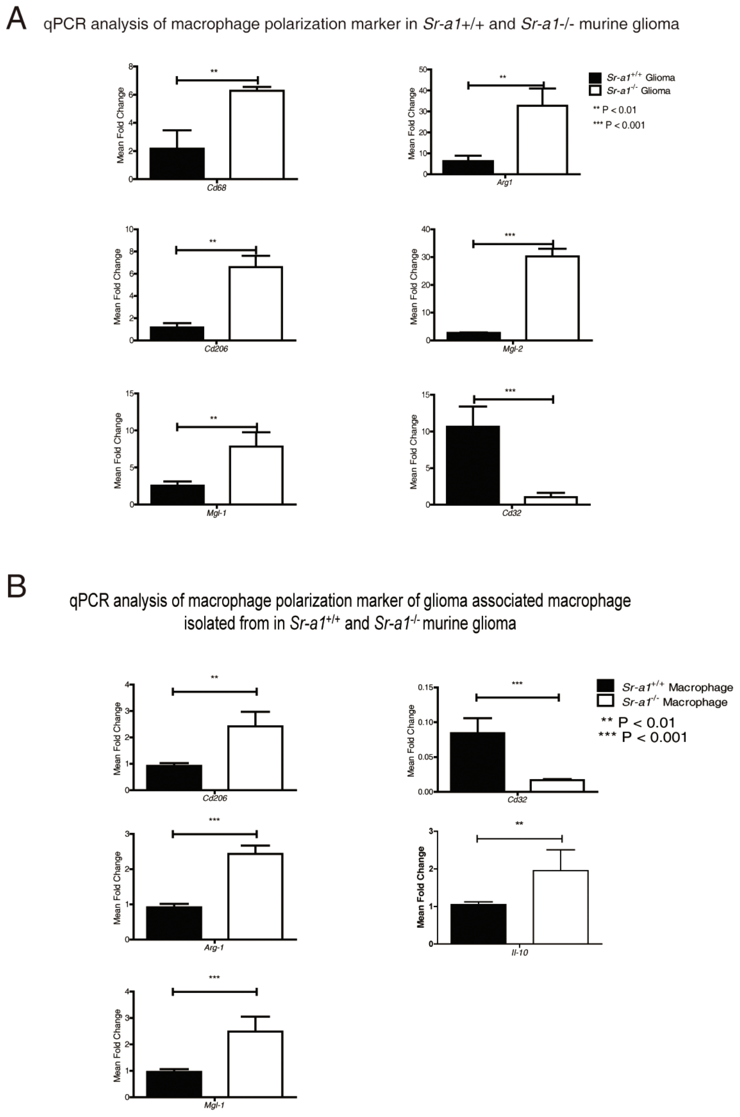

Supplementary Figure S4: qPCR analysis of macrophage/microglia polarization specific genes (M1-like: *Cd32*; M2-like: *Cd206, Arg-1, Mgl-1, Mgl-2* and *Il-10*) in murine orthotopic gliomas **A.** and glioma-associated macrophages **B.** (n = 6; *P<0.05; **P<0.01; ***P<0.001.)F4/80^{+} macrophages were isolated from both *Sr-a1*$^{+/+}$ and *Sr-a1*$^{-/-}$ murine orthotopic gliomas by FACS prior to qPCR.

A

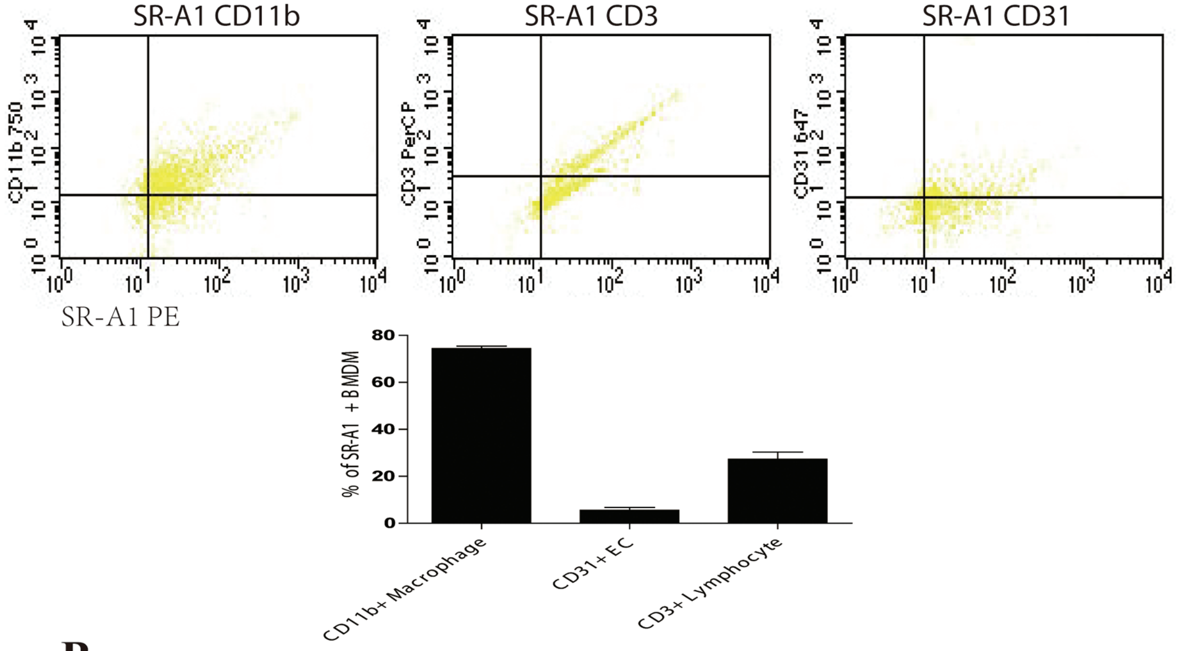

B

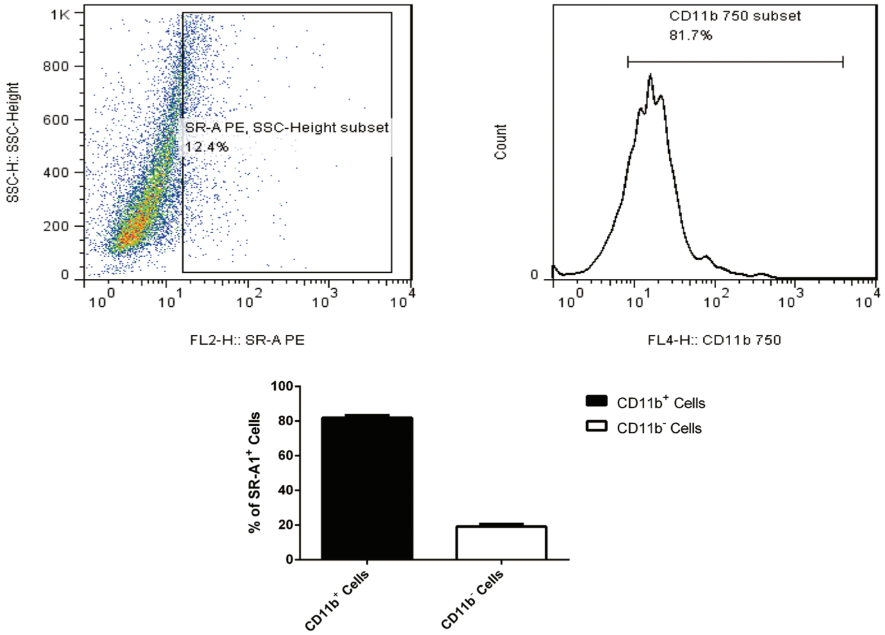

Supplementary Figure S5: Representative flow cytometry analysis of SR-A1⁺ cells in orthotopic glioma **A.** and macrophages **B.** Glioma samples were first gated for SR-A1-positive cells, then the gated cells were examined for expression of cell markers (macrophage: $CD11b^+$, lymphocyte: $CD3^+$, endothelial cell: $CD31^+$; n = 6). Macrophage samples were first gated for $CD11b^+$ cells; then the gated cells were examined for SR-A1 expression of (n = 6).

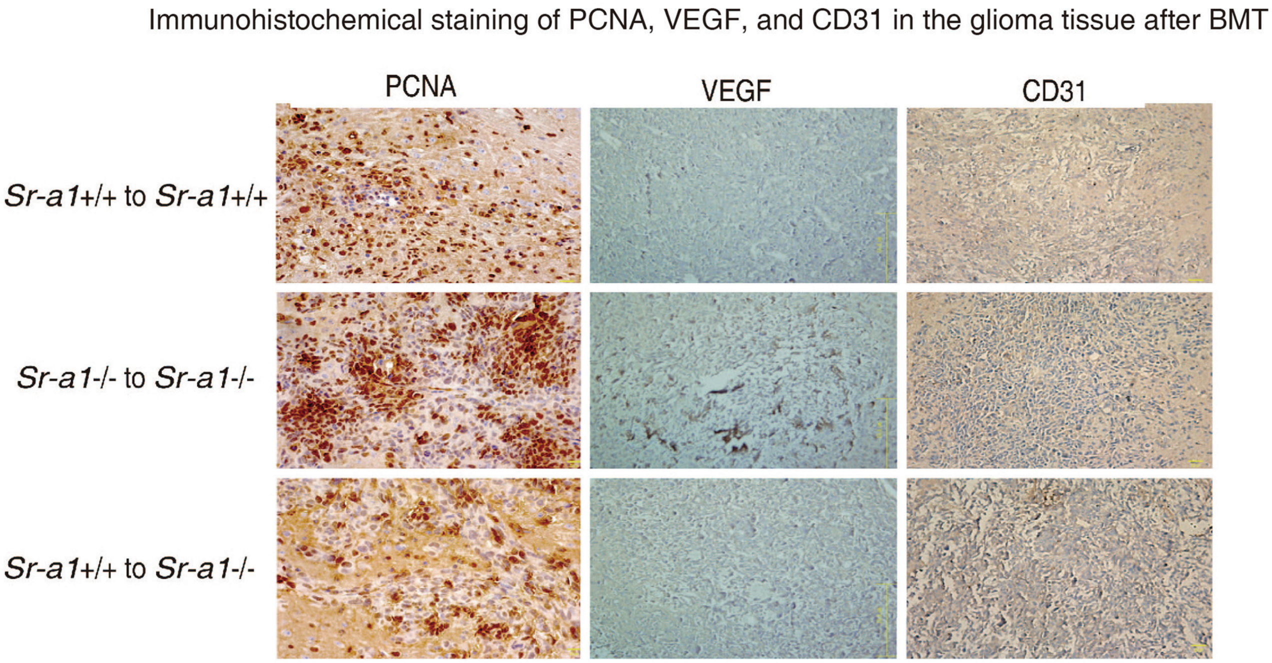

Supplementary Figure S6: Representative IHC staining of PCNA, VEGF and CD31 in brain tissue of bone marrow transplanted mice.

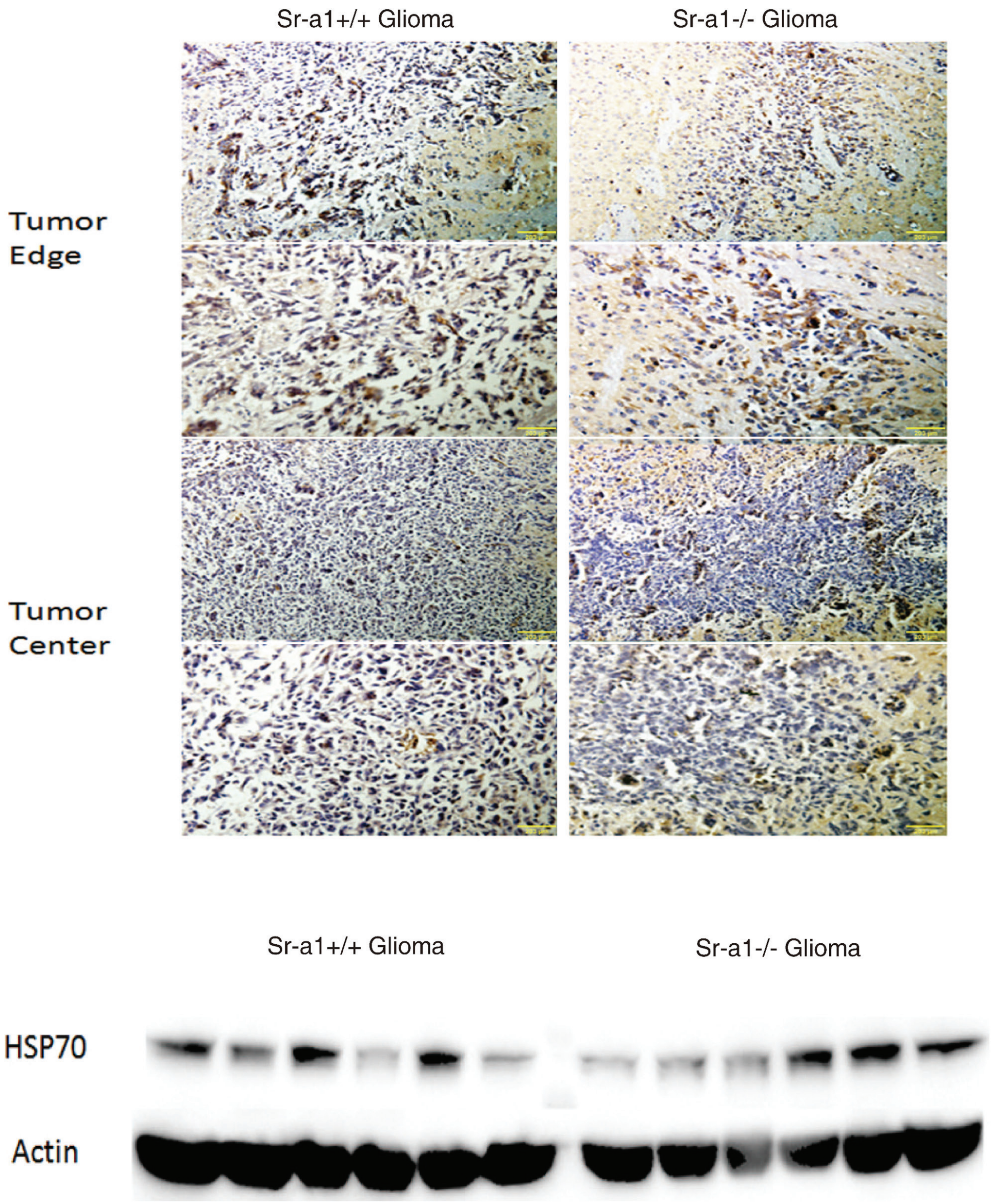

Supplementary Figure S7: Representative IHC staining and western blot analysis of HSP70 in murine orthotopic gliomas.

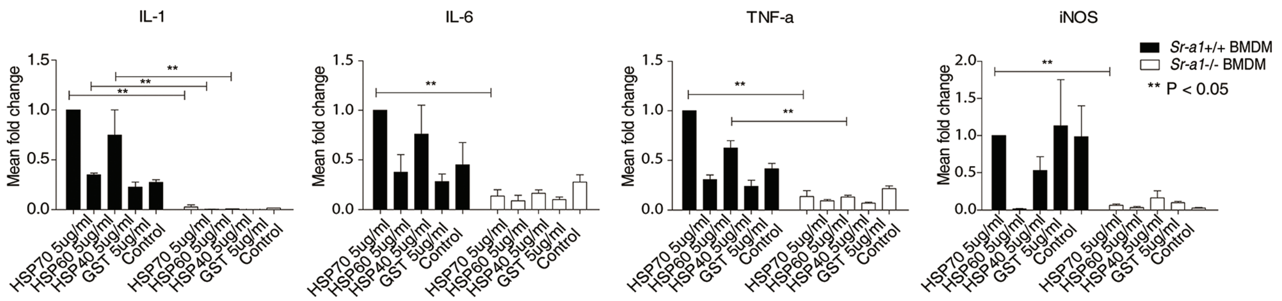

Supplementary Figure S8: qPCR measurements of cytokines in BMDMs induced by HSP family. BMDMs were treated with HSP70 (5 µg/ml), HSP40 (5 µg/ml), HSP60 (5 µg/ml) or GST (5 µg/ml) for 12 h (n = 6; *P<0.05; **P<0.01; ***P<0.001).

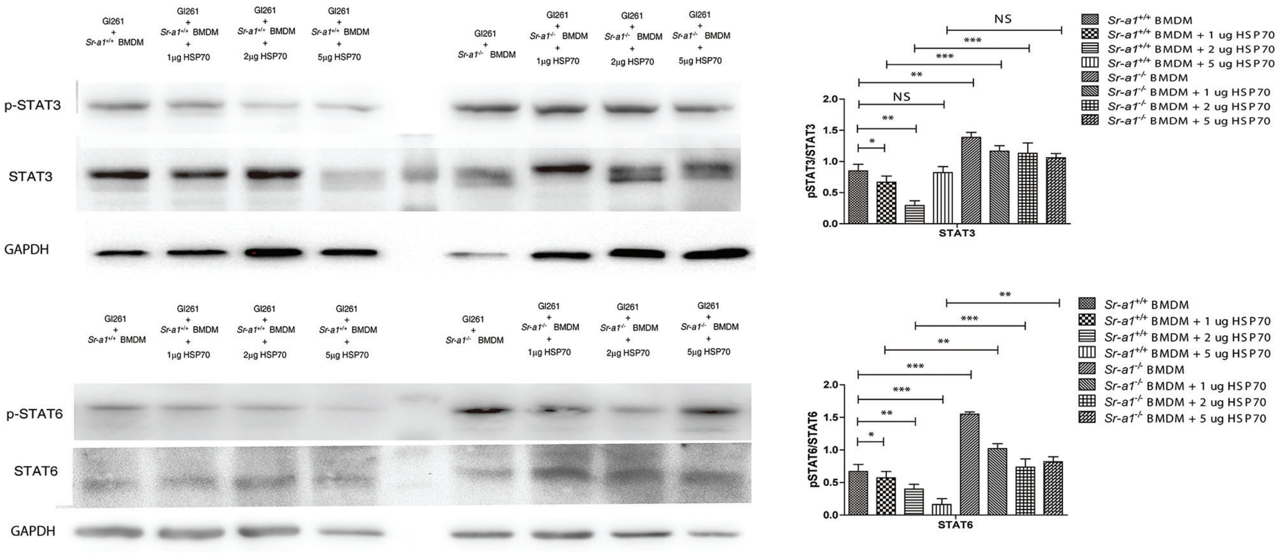

Supplementary Figure S9: Representative western blot of STAT3 and STAT6 in murine BMDMs treated with HSP70. Macrophages were treated with 1, 2, or 5 µg HSP70. Change in target protein was compared with total STAT3 and STAT6.
